# Symbiont-conferred immunity interacts with effects of parasitoid genotype and intraguild predation to affect aphid immunity in a clone-specific fashion

**DOI:** 10.1186/s12862-022-01991-1

**Published:** 2022-03-19

**Authors:** Samuel Alexander Purkiss, Mouhammad Shadi Khudr, Oscar Enrique Aguinaga, Reinmar Hager

**Affiliations:** 1 Division of Evolution, Infection and Genomics, School of Biological Sciences, Faculty of Biology, Medicine and Health, Manchester Academic Health Science Centre, The University of Manchester, Manchester, M13 9PT UK; 2 Departamento de Ingeniería, Facultad de Ciencias y Filosofía, Universidad Peruana Cayetano Heredia, Lima, Peru

**Keywords:** Intraspecific genetic variation effects, Inter-species indirect genetic effects, Intraguild predation, Indirect ecological effects, Pea aphid, Endosymbiont, Host–parasite system

## Abstract

**Background:**

Host-parasite interactions represent complex co-evolving systems in which genetic and associated phenotypic variation within a species can significantly affect selective pressures on traits, such as host immunity, in the other. While often modelled as a two-species interaction between host and parasite, some systems are more complex due to effects of host enemies, intraguild predation, and endosymbionts, all of which affect host immunity. However, it remains unclear how these factors, combined with genetic variation in the host and the parasitoid, affect host immunity. We address this question in an important agricultural pest system, the pea aphid *Acyrthosiphon pisum*, which shows significant intraspecific variability in immunity to the parasitoid wasp *Aphidius ervi*. In a complex experiment, we use a quantitative genetic design in the parasitoid, two ecologically different aphid lineages and the aphid lion *Chrysoperla carnea* as an intraguild predator to unravel the complex interdependencies.

**Results:**

We demonstrate that aphid immunity as a key trait of this complex host-parasite system is affected by intraspecific genetic variation in the parasitoid and the aphid, the interaction of intraspecific genetic variation with intraguild predation, and differences in defensive endosymbionts between aphid lineages. Further, aphid lineages differ in their altruistic behaviour whereby infested aphids move away from the clonal colony to facilitate predation.

**Conclusions:**

Our findings provide new insights into the influence of endosymbiosis and genetic variability in an important host-parasitoid system which is influenced by natural enemies of the parasitoid and the aphid, including its endosymbiont communities. We show that endosymbiosis can mediate or influence the evolutionary arms race between aphids and their natural enemies. The outcome of these complex interactions between species has significant implications for understanding the evolution of multitrophic systems, including eco-agricultural settings.

**Supplementary Information:**

The online version contains supplementary material available at 10.1186/s12862-022-01991-1.

## Background

Natural ecosystem dynamics and their evolution are driven by complex interactions of selective pressures on interacting species caused by both environmental and within-species genetic variation e.g. [1]. A textbook example extensively investigated at a theoretical and empirical level is the interaction between hosts and parasites. Here, the fitness of a parasite is dependent on its host and, despite a vast range of evolved anti-parasite responses, organisms continue to be successfully parasitised [2]. Parasites often manipulate their hosts to improve their fitness [3] through e.g. promoting predator avoidance responses in the host, increasing its likelihood of survival [4]. Particularly complex interactions often occur in parasitoidism, a process that represents aspects of parasitism and predation. Complete parasitoidism occurs when the larva of the parasitoid develops within its parasitised host as a parasite. This results eventually in host death through mummification, where only the exoskeleton remains after parasitoid emergence [5, 6]. An important example of a parasitoid is the hymenopteran endoparasitoid *Aphidius ervi* (Haliday), which is widely used as a biological control agent of the pea aphid *Acyrthosiphon pisum* (Harris) [7], in which it generally lays only one egg [6].

### Aphid resistance to parasitoids and the role of endosymbiosis

The co-evolutionary dynamics between antagonist species, such as an aphid host and its parasitoid, may drive evolution through a process of reciprocal adaptation and counter-adaptation, with selection for the development of resistance in the host and virulence traits in the parasitoid [8, 9]. The pea aphid shows considerable within-species variability in resistance to the parasitoid [10, 11], which is often conferred or mediated by specific microbial symbionts [12, 13]. The variation in resistance can be explained by different protective symbionts found in different aphid lineages [11, 13, 14]. Therefore, the natural enemy of the host is an enemy of the symbiont. Lineages of the pea aphid exist in clonally reproducing populations under temperate favourable conditions, and these clones carry vertically transmitted (secondary) facultative symbionts in addition to its (primary) obligate symbiont *Buchnera aphidicola* [15, 16]. The primary symbiont provides the aphid with nutrients that are lacking in its diet and that it could not otherwise produce [15]. Secondary symbionts have been implicated in various functions of aphid biology, including aiding in host-plant specialisation and particularly resistance to parasitoids [13, 15, 17].

### Indirect genetic and interspecies genetic effects

In addition to effects caused by facultative and obligate endosymbionts, within-species genetic variability will affect both focal and other interacting species and communities [18, 19] and can be highly influential in determining the interactions and ecology of host-parasitoid systems [20–22]. Further, indirect genetic effects theory outlines how the genotype of an individual can influence the phenotype of another individual (e.g. [23, 24]), of the same species (indirect genetic effect, IGE) or another species (indirect interspecies genetic effect, IIGE). In the aphid-parasitoid system, the parasitoid wasp *A. ervi* alters aphid behaviour by influencing where aphids move to die during wasp larval development [21]. Indeed, changes in aphid responses to the parasitoid are influenced by the genotype of the wasp [21], which thus represents an IIGE [21, 25, 26]. However, hosting different communities of defensive endosymbiotic bacteria, with associated symbiont-symbiont interactions and impact on the host [27, 28], can alter aphid phenotype and responses to challenges posed by natural enemies [29, 30]. Therefore, we need to consider the effects of both within-species genetic variability and their indirect effects when studying complex host-parasite systems.

### Complex ecoevolutionary dynamics and evolutionary arms-race

Following the Red Queen hypothesis, interacting species must constantly evolve to maintain their position as a form of an evolutionary arms race between the species [31], which may result in either reciprocal selective sweeps [32] or sustained genotype oscillations [33, 34]. In a host-parasitoid system, there is an arms race between host resistance (ability to survive the attack by the parasitoid) and parasitoid virulence (infectivity; the ability to overcome host defences) [31]. Most experimental examples of such co-evolutionary arms races [35] have demonstrated these as pairwise interactions between the parasitoid and its host. However, in natural ecosystems, parasitoids often do not operate in such pair-wise interactions as they are themselves interacting with other species that, for example, share aphid populations as prey [36, 37]. Therefore, host and parasite fitness will be significantly affected by the presence of a common enemy such as the aphid lion larva *Chrysoperla carnea* (Stephens). Aphid lions naturally have an advantage over parasitoid wasps. The aphid lion can consume both healthy and parasitised aphids (parasitoid puparia), an ecological process known as intraguild predation (IGP) [37]. This leads to reducing the availability of viable, healthy aphid hosts for the parasitoid and, concomitantly, an indirect reduction of parasitoid fitness [37, 38]. In response to parasitoidism, aphids are known to exhibit altruistic risk-taking behaviour by exposure to predators when parasitised, thus, in essence, sacrificing the diseased few for the benefit of the genetically identical population (clone) [39, 40]. Further, the bacterial symbiont, conferring a degree of immunity on the aphid host against its parasitoid enemy, adds another layer of complexity to the ecoevolutionary dynamics of the species interactions when intraguild predation occurs because the aphid lion is an enemy to the aphid host, the endosymbiont communities and the parasitoid. The complex interaction effects between within-species genetic variation of host and parasitoid when intraguild predation occurs may lead to ‘guild or diffuse co-evolution’ rather than pairwise co-evolution between two species [41–43], and may have important evolutionary implications for the pressures shaping aphid phenotype evolution in multi-trophic systems. To study such systems, a more complex approach is required, which, to date, has not been attempted. To address this gap in knowledge, we established a population of parasitoid *A. ervi* daughters using a half-sib quantitative genetic design, sensu Khudr et al. [21], and exposed two ecologically distinct lineages of the pea aphid *A. pisum*, with different defensive endosymbiont communities, to the intraspecific genetic variability effects of the parasitoid generated by the experimental design. We studied the immunity of the aphid host, with and without the presence of an intraguild predator (the aphid lion *C. carnea*)*.* We hypothesised that, subject to the intraguild predator, differences in defensive endosymbiont communities and differences in parasitoid genotype may differentially affect pea aphid reproductive success and behaviour in a lineage-specific manner.

## Results

In this study, we investigated the effects of parasitoid genotype provided with two aphid host conspecifics (N116 and Q1) that have different life histories and biotypes, on host fitness and behaviour under intraguild predation. As a measure of fitness, we focussed on the aphid immunity ratio (IR) that is the proportion of healthy aphids to the total population of healthy and parasitoidised individuals [mummified]), and host avoidance behaviour.

### Differences in immunity between the two aphid lineages (N116 and Q1)

The first step in our experiment was to establish the phenotypic differences between the aphid lineages, which showed that the two different clonal lineages are very different in the susceptibility to the parasitoid wasp, with the Q1 genotype being more susceptible than N116. Based on the known effects of defensive endosymbionts, we hypothesised that the two lineages differed in the defensive endosymbiont community they host. We, therefore, conducted an assay of the defensive (secondary) endosymbionts, which revealed that, unlike the Q1 genotype, N116 harboured different endosymbionts known to confer immunity to parasitoidism by the wasp *A. ervi* (Additional file 1, Molecular Analysis). We note that our objective was not to separate the effects of the endosymbionts on resistance from any potential intrinsic resistance to parasitoids, which would require removal of specific endosymbionts using antibiotic treatment. Of the 35 samples that were sequenced in the N116 clone, 26 were successful and contained a long enough sequence (590 bp to 1112 bp) to conduct a BLAST analysis. Of the 26 BLAST analysed samples, two were found to contain chimeric sequences and have been excluded, 13 samples matched with the known defensive secondary symbiont *Hamiltonella defensa* (99.19% to 99.87% identity). Furthermore, we also found nine samples were most closely related to *Fukatsuia symbiotica* (99% to 100% identity). Interestingly we also found that one sequence was most closely related to *Serratia symbiotica* (99% identity). For Q1, of the 35 samples sent for sequencing 23 were successful and of sufficient quality for BLAST analysis (420 bp to 967 bp). Here, 20 samples positively matched with the secondary symbiont *S. symbiotica* (99% to 100% identity), and we found no evidence for either *H. defensa* or *F. symbiotica in Q1.* (Additional file 1, Molecular Analysis).

### The effects of intraspecific genetic variation in the aphid and the parasitoid on aphid immunity when intraguild predation occurs

**Fig. 1:**
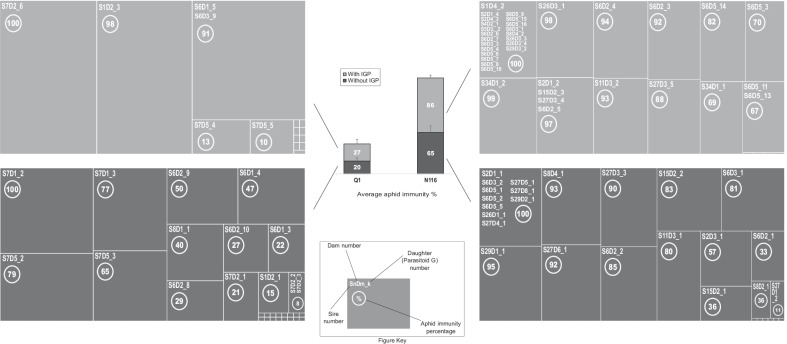
Aphid immunity subject to intraguild predation (IGP). The means (± SE) of IR (immunity ratio), representing aphid immunity to the parasitoid wasp, are shown centrally per aphid lineage with (light grey) and without (dark grey) IGP by the aphid lion. IR percentages, without IGP, are shown in dark grey; and in light grey with IGP. Each mean IR proportion is accompanied with detailed IR proportions for IR under the effect of the parasitoid genotype (daughters) that were the result of the Sire x Dam mating system (quantitative genetic design). In the absence of IGP sample sizes were as follows: n = 30 parasitoid daughters in the case of N116, and n = 30 in the case of Q1. In the presence of IGP: n = 43 parasitoid daughters in the case of N116, and n = 15 in the case of Q1. In total, there were 118 parasitoid daughters. The smallest uniform rectangles shown in the detailed IR illustration refer to IR of 0% i.e., lack of immunity

Having established differences in endosymbiont communities, we then proceeded to our full experiment in which we focussed on aphid immunity as defined above. As shown in Fig. 1, the overall average immunity ratio (IR) of N116 was ~ 65% in the absence of IGP, which increased to 86% when IGP was present. By contrast, the average IR of Q1 was ~ 20% (IGP absent), which increased to ~ 27% when the IGP was present. Thus, IR in N116 was 3.25 times higher than in the Q1 lineage without IGP, and ~ 3.2 times higher with IGP. The IR was significantly affected by parasitoid sire (F_(13,45)_ = 36.12, P = 0.0006) and dam parasitoid genotype (F_(7,45)_ = 23.92, P = 0.001), parasitoid genotype (F_(36,45)_ = 174.46, P < 0.0001), the interaction between the two (F_(4,45)_ = 11.23, P = 0.024), and the three-way interaction (parasitoid genotype x aphid lineage x IGP; F_(11,45)_ = 32.5, P = 0.0006). We recorded total immunity (IR = 100%) to the parasitoid in 10 out of 30 cases for N116 versus only one case out of 30 for Q1 when IGP was absent, and 24 cases out of 43 for N116 versus only one case out of 15 for Q1 when IGP was present. Conversely, for lack of immunity (IR = 0%), there were six cases out of 30 for N116 versus only 16 cases out of 30 for Q1 when IGP was absent, and four cases out of 43 for N116 versus only nine cases out of 15 for Q1 when IGP was present.

The parasitoid was less successful in sequestering aphids as puparia when the intraguild predator was present, and that was clearly pronounced in the N116 aphid lineage, which harboured defensive symbionts, in contrast to Q1.

### Aphid altruistic mummification behaviour

**Fig. 2:**
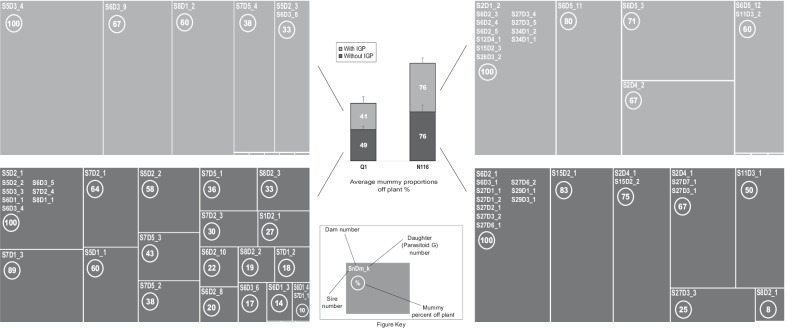
Proportions of mummies off-plant subject to intraguild predation (IGP). Percentages of aphid mummies (means ± SE) recorded off the plant are shown for N116 and Q1 exposed to the effect of the parasitoid genotype (daughters) in the absence of IGP (dark grey) and the presence of IGP (light grey). Sample sizes were as follows: In the absence of IGP: n = 20 parasitoid daughters in the case of N116, and n = 29 in the case of Q1. In the presence of IGP: n = 19 parasitoid daughters in the case of N116, and n = 13 in the case of Q1. In total there were 81 parasitoid daughters. The smallest uniform rectangles shown in the detailed illustration refer to the off-plant mummy proportion of 0%. The parasitoid genotype (daughters) were the result of the Sire × Dam mating system (quantitative genetic design)

As shown in Fig. 2, the average off-plant proportions of mummified aphids were almost identical in the case of N116 (~ 76%) with and without IGP. By contrast, for Q1 the average percentage was ~ 49% in the absence of IGP, and ~ 45% in presence of IGP. Parasitised N116 individuals mummified ~ 1.55 times more than Q1 when the IGP was absent, and ~ 1.69 times more in the presence of IGP (Fig. 2). The proportion of mummies off-plant were significantly affected by parasitoid sire (F_(12,35)_ = 31.19, P = 0.002), parasitoid genotype (F_(16,35)_ = 35.97, P = 0.003), IGP (F_(1,35)_ = 4.43, P = 0.035), aphid lineage (F_(1,35)_ = 9.4, P = 0.002), the interaction (parasitoid genotype x aphid lineage; F_(2,35)_ = 10.03, P = 0.007), and the interaction (parasitoid genotype x aphid lineage x IGP; F_(12,35)_ = 21.93, P = 0.038). The mummification of N116 and Q1 showed similar relative proportions with or without IGP, but the numbers of mummies off and their proportions off plant were more varied, and more apparent in N116.

## Discussion

We established that the two different clonal lineages are very different in their responses to the parasitoid wasp, with the Q1 genotype being more susceptible than N116. Furthermore, the impact of IGP on aphid immunity/parasitoid virulence was more notable in N116. *Hamiltonella defensa is* a defensive secondary symbiont found throughout pea aphid lineages [44] and reported to provide immunity to parasitoidism by stopping the development of the *A. ervi* larva, and hence rescuing the aphid host [13, 44]. The level of conferred immunity can vary substantially by different strains of *H. defensa* and the spread of the endosymbiont may rapidly increase, in experimental populations, with exposure to parasitoid wasps [45, 46]. Variation in protection is further influenced by the presence or absence of infection of the bacteria with different bacteriophages called APSEs [45]. These bacteriophages are thought to encode putative toxins that function in the specific defence against *A. ervi* [14, 44], which, however, we did not investigate in this study. Moreover, unlike Q1, N116 harboured *F. symbiotica* previously referred to as the X-type or PAXS symbiont, that, when found in association with *H. defensa,* provides high levels of resistance to *A. ervi* [47–49]; *F. symbiotica* and *H. defensa* were previously reported in the N116 lineage [49]. Interestingly, we found that N116 harboured an additional endosymbiont species (*S. symbiotica*), which is another known symbiont that may provide some resistance against parasitoids [13, 15, 46, 50, 51]. In a striking contrast, Q1 harboured *S. symbiotica* only. Isolates of both *S. symbiotica* and *H. defensa* have been shown to confer resistance to parasitoid wasps in the pea aphid, reducing successful parasitism by 23% and 42%, respectively [13, 15]. Moreover, the occurrence of superinfected aphid clones (carrying multiple inherited symbionts), has been noted despite the apparent costs to aphid fecundity [50].

This supports our findings of the immunity differences between Q1 and N116, given the lack of evidence of strong intrinsic immunity in pea aphids through encapsulating parasitoid eggs [11, 13, 15, 16]. The immunity to the parasitoid *A. ervi* is usually conferred by defensive endosymbiotic bacteria as demonstrated by our findings, especially since the pea-aphid genome lacks essential immunity genes [52]. Furthermore, aphids superinfected with *H. defensa* and *F. symbiotica* are known to have very high levels of resistance against *A. ervi*, up to 100% in some clones [29, 30, 48, 49, 53]. The immunity of N116 was, therefore, boosted by the presence of this combination of endosymbionts [15, 46–51].

As such, it can be argued that the effect of the aphid ‘genotype’ in this work is more than the effect of the genotype alone, as it also includes the indirect factor of defensive endosymbiosis in association with the lineage. This interpretation receives support from Oliver et al. [13] who demonstrated that the presence of the heritable defensive secondary symbiont is more important than the aphid genotype regarding the immunity of pea aphids to the parasitoid *A. ervi*. As such, the symbiosis with defensive bacteria alters the outcome of the interaction between the parasitoid and the aphid host and thus should be considered an important indirect ecological effect in this system [54]. We advocate that the indirect ecological effect of endosymbiosis influenced the outcome of the effect of parasitoid genotype on the reproductive success of its aphid hosts. Clearly, the presence of co-existing defensive endosymbionts proved beneficial for N116, but see [28]. The strong and intimate interaction between the aphid host and its parasitoid may be influenced by genetic variation in the traits related to the interaction of the species involved, meeting one of the fundamental criteria for co-evolution in a host-parasitoid system [12]. Although the N116 pea aphid is one of the lineages with a known association with *H. defensa* [55]*,* to the best of our knowledge, we are the first to empirically test the immunity in this lineage when an intraguild predator is present.

Aphid mummification off-plant, away from the healthy clonal population, has been interpreted as altruistic behaviour because it leads to an increased predation risk for parasitised aphids but a reduction in successfully eclosing parasitoid wasps [39, 40, 56]. Our results suggest that N116 showed a consistent propensity to desert the host plant when parasitised. Yet, the ecological effect of IGP on such a propensity was negligible in N116. Conversely, Q1 showed less altruistic behaviour than N116, with a bigger margin of difference (8%) between the absence and presence of the aphid lion, when compared to N116. Under the life-dinner principle [57], changes in aphid population and altruistic behaviour may lead to changes in the parasitoid host-manipulative tactics and virulence, such that decreased aphid altruistic behaviour may reduce the parasitoid loss inflicted by an intraguild predator (which shares the aphid as prey with the parasitoid). Thus, parasitoid wasps alter the behaviour [21] as well as the internal environment of the parasitised aphid to make it more favourable for wasp development and survival [58]. Our findings suggest that pea aphid responses (including the location of mummies) to parasitoidism accompanied by IGP, may depend on the within-species genetic variability in both the aphid and the parasitoid, as well as the indirect effect of absence or presence of complex defensive endosymbiosis.

Having more aphid mummies (wasp puparia) farther from the core of the mother clone is assumed to increase aphid inclusive fitness, but this altruistic change in mummy position is likely to be cost-sensitive and context-dependent [21, 56, 59–61]. It is worthy of note here that parasitoid wasps may be able to differentiate between infected and uninfected aphids with the facultative endosymbiont *H. defensa*, thought to be the result of a decreased production of a major component of the aphid alarm pheromone (EBF) [58]*.* The alarm pheromone is secreted from cornicles when the aphids are attacked, and when aphids detect the pheromone they move away from the source, with some even dropping from the plant altogether [58]. This potential of *A. ervi* to differentiate between aphids infected with *H. defensa* and those that are not is demonstrated by an increased occurrence of superparasitism in the infected aphids. Superparasitism occurs when more than one egg is oviposited into the same aphid host and, under normal conditions, this behaviour is usually considered maladaptive as it results in siblicide [62]. Interestingly, the presence of *H. defensa* in a host aphid may have further implications for the plant-aphid-parasitoid system as it alters the behaviour of the parasitoids [14, 58]. Vorburger and Rouchet [34] suggested that there may be selection for local adaption by parasitoids to certain strains of *H. defensa,* but this remains in need of further investigation [34]. This implies that the interaction between the aphid (including the defensive symbiosis) and the parasitoid is highly context-dependent as shown in our study. Moreover, *H. defensa* is also implicated in changing aphid defensive behaviour against parasitoids [63] and in attenuating the release of herbivore-induced plant volatiles that attract parasitoid wasps [64]. This further highlights the importance of symbionts in the interactions between species [14, 64] such that defensive symbionts are reported to have far-reaching ecological effects on aphid-parasitoid communities [65]. The rate of evolution of host resistance to parasitoids, as well as the infectivity (virulence) of parasitoids will be subject to the impacts of internal defensive symbionts [61, 66; see also 67] and external factors (e.g. intraguild predators) [36, 37]. Altogether, these are constituents of an ongoing evolutionary arms-race [35, 41–43] that will depend on the levels of variation present in the populations and the associated fitness costs of the involved traits [61, 67]. This is in line with the extensive effects of intra-specific genetic variation of one species on other species beyond the individual or population levels [18, 19]. Vorburger and Perlman [68] proposed that defensive endosymbionts can be active in a three-way interaction that may alter the ecoevolutionary reciprocity of selection in the host-parasitoid system. Our findings show that endosymbiont effects can be involved in higher-level interactions, in our case the interaction of endosymbionts, the aphid, the parasitoid, and the intraguild predator.

Our study has demonstrated the complex nature of the interaction between two lineages of a scientifically as well as economically important agricultural pest and the genotype of its parasitoid subject to the effects of intraguild predation by the aphid lion. Our findings imply that having defensive endosymbiosis may contribute to aphid survival and reactions to differential parasitoid virulence that appear to be context-dependent. The influence of the presence of the intraguild predator varied across parasitoid genotypes and aphid lineages. We demonstrate the need to consider the effects of intra-specific genetic variation in host-parasitoid systems together with the ecological effects brought about by defensive endosymbiosis and other natural enemies of the aphid across trophic levels. This will help untangle the complexity of these interactions and hence design effective biological controls in agro-ecosystems.

## Methods

### Study organisms

#### Pea aphids and defensive endosymbiont

Two clonal lineages of pea aphid were selected for the experiment, N116 and our Q1 isolate. The N116 aphid is of the biotype (K) as it was originally isolated from alfalfa *Medicago sativa* (L.) by Dr Julia Ferrari in Berkshire UK [40]. It has been a laboratory lineage for *ca.* 10 years and was provided to us by Dr Colin Turnbull of Imperial College London. Q1 is of the biotype (G) [69], which was established from one female of a population colonising pea plants (*Pisum sativum* L.) isolated from the quadrangle garden of the Faculty of Biology, Medicine and Health, University of Manchester. N116 is reported to have the heritable defensive endosymbiont *H. defensa* [55] that confers relative immunity to parasitoidism. By contrast, we established that Q1 was highly susceptible to being parasitoidised. As specified in the molecular analysis below, we surveyed the endosymbiont communities in each of the lineages. N116 and Q1 are ecologically distinct, derived from different geographic locations, have distinguishable life histories and susceptibility to the parasitoid wasp. They are, therefore, a good representative of the within-species genetic variability in the pea aphid. The aphids were reared on faba bean *Vicia faba* var minor (Harz) plants obtained from a local supplier, Manchester, UK, and maintained at 22–24 °C with a photoperiod of 16 h (light): 8 h (dark). Under temperate mesic conditions, aphids reproduce through parthenogenesis resulting in populations of genetically identical individuals.

#### Parasitoid wasp *A. ervi*

We purchased 250 mummies of aphids harbouring *A. ervi* juveniles from Koppert Biological Systems (UK). Unlike the non-parasitic males, the females of this solitary koinobiont parasitoid wasp are an efficient natural enemy and biocontrol agent of pea aphids [37, 70]. The female oviposits one egg in the viable aphid host. Subsequently, a larva hatches and parasitises the host consuming it internally whilst the parasitoid juvenile pupates, then develops into an adult that ecloses from the dead body of the host to resume the life cycle. Immediately upon their arrival, we separated the mummies into multiple 90 mm petri dishes, each dish containing a small ball of dental cotton, approximately 20 mm in diameter, which was saturated in 10% sucrose solution. The petri dishes were kept in the fridge at 10 °C to slow the rate of eclosion from the aphid mummy (i.e. the wasp puparium). The petri dishes were taken from the fridge hourly and checked for the eclosion of wasps; the sex of the emergent wasp was observed; if all the individuals were of the same sex, then they could be used in the next stage of the experiment. The females were always isolated and kept separately from the males to ensure the females were virgins prior to mating according to the quantitative genetic design explained below.

#### Intraguild predator *C. carnea* larva

The intraguild predator in our experiments was the aphid lion larva. The larvae were purchased from Ladybird Plant Care (UK) in tubes of approximately 300–500 individuals. The tube was emptied into a plastic container that contained some plant shoot parts with aphids as a provision and then kept in the fridge at 5 °C until they were needed; this was to slow the rate of metabolism and prevent the larvae from cannibalising each other. The larvae were used within 48 h of delivery or they were disposed of. As the wasps take ~ 11 days to emerge from the mummies, the aphid lion larvae (1st instars) were ordered so that they would arrive on day 10 ready to be used where applicable in the experiment as described below.

### Experimental design

**Fig. 3:**
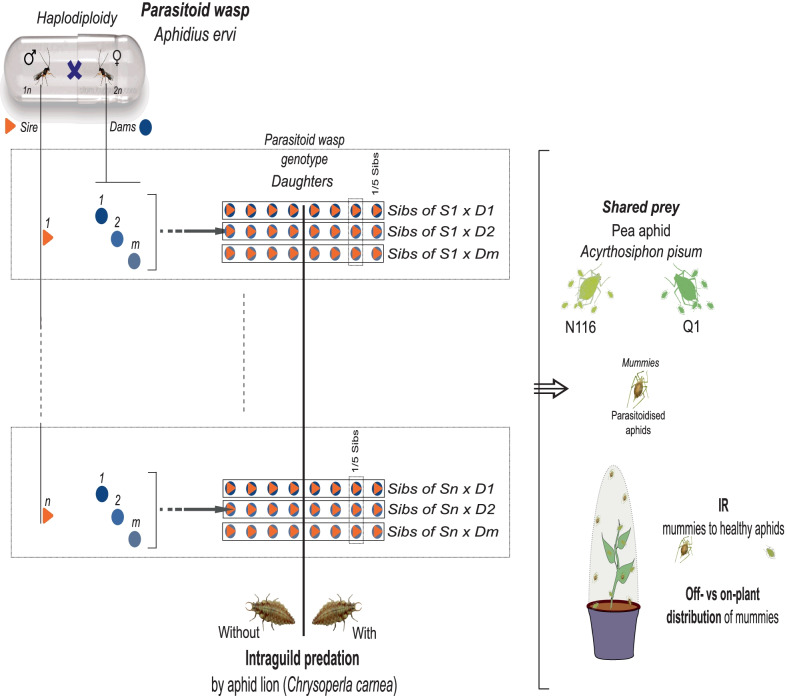
Experimental design. The diagram shows the full experimental design, with n sires being mated to at least three dams (D1 – D*m*). The sire × dam mating groupings produced the intraspecific genetic variability in the parasitoid (genotype/daughters [sibs, and half-sibs]). Overall, there were 119 parasitoid daughters. Each group of daughters of the (S1 – S*n*) combinations was then split into two populations, with one (n = 73) being provided with pea aphids of the N116 lineage as a provision, while the other (n = 45) provided with pea aphids of the Q1 lineage. Each of these populations where further split into two groups, with one group exposed to intraguild predation by the aphid lion larva (n = 43, in the case of N116, and n = 15 in the case of Q1) and the other group not (n = 30, in the case of N116, and n = 30 in the case of Q1). The effects of parasitoid and aphid genetic variability (with and without the aphid lion) on aphid immunity ratio (IR) were investigated in microcosms. IR is the proportion of healthy aphids (non-mummified i.e. unparasitoidised) after 11 days of exposure to the parasitoid genotype relative to the entire population of aphids (healthy and mummified) per aphid lineage

Haplodiploidy is the sex-determination system in the Hymenopteran parasitoid wasp *A. ervi*, meaning that males are the result of unfertilised eggs and hence haploid (1n), while females are diploid (2n) since they are produced from fertilised eggs [71]. Based on Khudr et al. (2013) [21], we mated randomly selected 34 male wasps (sires) with randomly selected female wasps (dams) to establish a quantitative genetic half-sibling design. Each of the sires was mated with a minimum of three dams, dependent on wasp availability right after their eclosion. We thus established sire-dam groups. Before the wasps were mated, they were isolated into Eppendorf tubes and inspected using a magnifying glass to observe abdomens and determine their sex; the female’s abdomen ends with a pronounced point (ovipositor) while the male’s abdomen is more rounded. The wasps were then put into the same tube by opening both tubes and putting them end to end. Once both wasps (sire and dam) had moved into the same tube, it was sealed with a small piece of foam. The mating wasps were monitored carefully until they completed copulation to ensure the corresponding sire inseminated the assigned dams. Copulation was checked to have occurred within two hours of eclosion. If copulation did not happen, the female wasps were disposed of because of the short window of time during which the otherwise arrhenotokous parthenogenetic female wasp will be usually receptive to mating [21, 71]. Once copulation was completed the foam was removed, the tubes were placed end to end, and we waited for the wasps to enter separate tubes before closing the lids and labelling the sire with its unique number (S1 – S*n*), and the dams with the number of the associated sire they mated with plus their own unique number in order of mating (*e.g.* S1 D1 – S*n* D*m*). Figure 3 illustrates the experimental design.

Once mated, the inseminated dams were placed in their respective microcosms. The microcosms were constructed by removing the ends of a 2-L PVC bottle and attaching one end to the plant pot and covering the other with a fine nylon mesh (‘Non-Fray’, Insectopia, UK). Each microcosm contained a 3-week-old broad bean plant that had been infested with 30 third instars of N116 just before putting the wasp into the enclosure. To release the dam into the microcosm the top section was held in place over the plant (leaving a small gap on one side), the lid of the tube was opened and sealed with the end of a finger and then the tube was passed through the gap onto the soil. Once the inseminated wasp was inside the microcosm, the top section of the microcosm was secured to the plant pot using 48 mm wide polypropylene tape. The microcosms were placed, evenly spaced, into large trays, containing a shallow layer of water, in the growth chamber for eleven days. The conditions in the chamber were 22–24 °C with a 16 h (light):8 h (dark) photoperiod; the water level in the trays was maintained and the positions of the microcosms on the trays were randomised every other day. On the eleventh day, the microcosms were taken from the growth chamber, opened, and all the mummies present were removed from the plant and inner surfaces of the microcosm using a fine damp paintbrush. Each mummy was placed in a separate 35 mm petri dish that contained a small ball of dental cotton (approximately 10 mm in diameter) saturated with 10% sucrose solution and labelled with the associative sire-dam number. The petri dishes were left at room temperature on the lab bench and left until we observed eclosion. Once the progenies (sib and half-sib daughters denoting the intraspecific genetic variability of the parasitoid) had emerged from the aphid mummies, they were individually introduced into a microcosm with a 3-week-old faba bean plant that had been infested with 30 third instars of N116. The microcosms were sealed, and each of the introduced daughters (i.e. parasitoid genotype) was given 11 days to parasitise the provided aphid population leading to the production of mummies. We then censused the aphid population in each microcosm (mummified and healthy) and recorded the positions of the mummies off-plant versus on-plant. The whole procedure was simultaneously repeated for the Q1 lineage. As such, the wasp daughters (parasitoid genotype) represented the intraspecific genetic variation effects in the parasitoid wasp, whereas the within-species genetic variation in the pea aphid host was represented by the inclusion of the N116 and Q1 lineages.

The remainder of the generated parasitoid daughters were used to test the effect of the presence of the aphid lion as an intraguild predator (IGP) on aphid traits. After the introduction of the aphids (N116 or Q1) followed by the parasitoid daughter into the microcosm, as explained above, an aphid lion second-instar larva was transferred into the microcosm, on a fine paintbrush, onto the soil a few minutes after the wasp was added. The daughters (parasitoid genotype) that arose from each of the sire × dam mating groupings were numbered and then split randomly into one of two groups: without IGP (*i.e.* IGP absent) or with IGP (*i.e.* IGP present). Once the microcosm set up was completed, they were sealed and placed back into the growth chamber for eleven days at 22–24 °C with the 16 h:8 h photoperiod as above. The microcosms were randomised in the chamber and checked to ensure that they had enough water every other day. On the eleventh day, the microcosms were once again removed from the growth chamber, opened and the data were recorded. We recorded the total number of healthy aphids (non-mummified), the total number of mummies, and the distribution of the mummies within the microcosm (on versus off-plant), (Fig. 3). We were unable to create a fully factorial design with two aphid lineages and the presence or absence of a predator for each dam/sire combination. The differential survival in this multispecies system combined with the nature of the quantitative genetic design, and keeping all the parthenogenetic aphids at the same age, led to unbalanced sample sizes for a given aphid lineage, which, nevertheless, is sufficiently powered for the number of replicates. Overall, we were left with 118 parasitoid genotypes (daughters). Daughters were split into two groups, with one (n = 73) being provided with pea aphid N116 as provision, while the other (n = 45) was provided respectively with pea aphid Q1. Each of these two populations were further split into two groups, with one group exposed to intraguild predation by the aphid lion larva (n = 43, in the case of N116, and n = 15 in the case of Q1) and the other group not (n = 30, in the case of N116, and n = 30 in the case of Q1).

#### Molecular analysis

Healthy aphids from each microcosm were preserved in a cryogenic tube at − 195 °C, at The University of Manchester liquid nitrogen sample storage facility, for molecular analysis. The identification of the bacterial symbionts in the two lineages of pea aphid consisted of two parts: (1) the use of diagnostic PCR to confirm the presence or absence of the defensive symbiont *H. defensa,* and (2) 16 s rRNA gene sequencing for the identification of other symbionts. The aphid samples were surface-sterilised [72], then the DNA was extracted using ‘Qiagen DNAEasy Blood and Tissue Kit’ small insect supplementary protocol [72]. As the aphids are soft-bodied insects, we altered step 1 of the protocol slightly, rather than freezing them in liquid nitrogen and grinding them up in a pestle and mortar they were homogenised in a sterile microcentrifuge tube using a sterile disposable microcentrifuge tube homogenisation pestle. In step 3, the lysis time was increased from three to six hours and the rest of the protocol was followed with no further modifications. Subsequently, we ran a Diagnostic PCR [73]; the PCR reactions were visualised on a 1% agarose gel with SafeView Nucleic Acid Stain with Bioline HyperLadder™ 1 kb. Afterwards, we ran 16 s Gene Sequencing for a total of 70 samples (35 Q1 and 35 N116), which were sent for sequencing using GATC Biotech’s T7 sequencing primers. Once we had received the sequence data, both the vector sequences and the parts of the sequences that contained bases that were below the confidence threshold were removed. The sequences were then analysed using the NCBI ‘standard nucleotide BLAST’ (megablast) and the Nucleotide collection (nr/nt). The most closely related bacteria were selected based on the blast output and where they fall on the resulting distance tree of the results (Additional file 1, Molecular Analysis).

### Statistics

The data on the parasitoid genotype with and without IGP were pooled because this enabled us to investigate the influence of the IGP on the outcome of the parasitoid genotype effect on aphid fitness (in terms of immunity to the parasitoid) and the behaviour of the aphid lineages. All statistical analyses were conducted using R [74] via RStudio [75]. Firstly, we tested the effects of parasitoid and aphid genetic variability in the absence or presence of IGP on aphid immunity ratio (IR: the proportion of aphids that was non-mummified [unparasitoidised] after 11 days of exposure to the parasitoid genotype relative to the entire population of aphids [healthy and mummified] per aphid lineage per microcosm). We applied a generalised linear mixed effect model (GLMMER1) with a Poisson family, R packages ‘car’ [76] and Ime4 [77]. The parsimonious model included the following explanatory variables as fixed effects: (1) sire (14 levels), (2) dam (8 Levels), (3) parasitoid genotype (daughter identity as per their sire × dam mating grouping that was the product of the quantitative genetic design; 118 daughters in total representing the parasitoid intraspecific genetic variation effect), (4) aphid lineage (two levels [N116, Q1]), (5) the interaction (parasitoid genotype × aphid lineage), (6) the interaction (parasitoid genotype × aphid lineage × Intraguild Predator presence (IGP [No, Yes])). The microcosm was modelled as a random effect.

Secondly, we analysed aphid behaviour as the proportion of aphid mummies off-plant relative to the total number of mummies in the microcosm. We used a generalised linear mixed-effect model (GLMMER2) with a Poisson family. Again, the parsimonious model included the following explanatory variables as fixed effects: (1) sire (13 levels), (2) parasitoid genotype (daughters’ identity as per their sire × dam mating grouping that was the product of the quantitative genetic design; 81 daughters in total representing the parasitoid intraspecific genetic variation effect), (3) aphid lineage (two levels [N116, Q1]), (4) Intraguild Predator (IGP) presence (two levels [No, Yes]), (5) the interaction (parasitoid genotype × aphid lineage), (6) the interaction (parasitoid genotype × aphid lineage × IGP). The microcosm was modelled as a random effect.

## Supplementary Information

**Additional file 1.** Details of the molecular analysis 16s rRNA sequences used in the Blast analysis.

## Data Availability

The datasets generated and/or analysed during the current study are available in the Figshare repository, https://figshare.com/s/96df54283ac09ebd39cd. Gene Bank accession numbers: N116 aphid endosymbionts: MW979375 to MW979398; Q1 aphid endosymbionts: MW971996 to MW972018.

## Abbreviations

BLAST: Basic Local Alignment Search Tool
Car: Companion to applied regression
D: Dam
EBF: E-beta-farnesene
GLMMER1: Generalised linear mixed effect model 1
GLMMER2: Generalised linear mixed effect model 2
IGE: Indirect genetic effect
IIGE: Indirect interspecies genetic effect
IGP: Intraguild predation
IR: Immunity ratio
Ime4: Linear mixed-effects models using 'Eigen' and S4
nr/nt: Non-redundant nucleotides
PCR: Polymerase chain reaction
PVC: Polyvinyl chloride
rRNA: Ribosomal ribonucleic acid
S: Sire
