# Supplementary material for: Symbiont-conferred immunity interacts with effects of parasitoid genotype and intraguild predation to affect aphid immunity in a clone-specific fashion
Source: BMC Ecol Evol. 2022 Mar 19;22:33. doi: 10.1186/s12862-022-01991-1 (PMC8934488; doi:10.1186/s12862-022-01991-1)
Supplement: Supplementary file 1 — Additional file 1. Details of the molecular analysis 16s rRNA sequences used in the Blast analysis. [file 12862_2022_1991_MOESM1_ESM.docx]

**Additional Information**

**Symbiont-conferred immunity interacts with effects of parasitoid genotype and intraguild predation to affect aphid immunity in a clone-specific fashion**

Samuel Alexander Purkiss^1*^, Mouhammad Shadi Khudr^1*^, Oscar Enrique Aguinaga^2^, Reinmar Hager^1^

^1^ Division of Evolution, Infection and Genomics; School of Biological Sciences; Faculty of Biology, Medicine and Health; Manchester Academic Health Science Centre; The University of Manchester, M13 9PT, Manchester, UK

^2^ Departamento de Ingeniería, Facultad de Ciencias y Filosofía, Universidad Peruana Cayetano Heredia, Lima, Peru

Corresponding author: Reinmar Hager

*Equal contribution.

## Molecular Analysis

**Table S1. Primers used in this study.** The table shows the target symbiont, the respective target genes with their primer names, followed by the sequence (5’-3’) and the references given below the table. Note that primers used in diagnostic 16s PCR were used to only confirm presence or absence of H. defensa.

| Target symbiont | Target gene | Primer name | Sequence (5’-3’) | References* |
| --- | --- | --- | --- | --- |
| **Primers used in diagnostic 16s PCR** | | | | |
| *H. defensa* | 16s rRNA | 10F | AGTTTGATCATGGCTCAGATTG | 1 |
| *H. defensa* | 16s rRNA | 16s- T419R | AAATGGTATTSGCATTTATCG | 1 |
| **Primers used in PCR reactions for 16s gene sequencing** | | | | |
| Universal | 16s rRNA | fD1 | AGAGTTTGATCCTGGCTCAG | 2 |
| Universal | 16s rRNA | rP2 | ACGGCTACCTTGTTACGACTT | 2 |

* References: 1) Henry, L., Peccoud, J., Simon, J., Hadfield, J., Maiden, M., Ferrari, J. and , H., 2013. Horizontally transmitted symbionts and host colonization of ecological niches. Current Biology, 23(17), pp.1713-1717. 2) Weisburg, W., Barns, S., Pelletier, D. and Lane, D., 1991. 16S ribosomal DNA amplification for phylogenetic study. Journal of Bacteriology, 173(2), pp.697-703.

## Gel extraction

Once the universal 16s PCR reactions were completed [*95°C 5 mins, (95°C 30s, 60°C 30s, 72°C 30 sec) x 40 and a final extension of 72°C 7 mins*] they were visualised on a 1% agarose gel with SafeView Nucleic Acid Stain with Bioline HyperLadder™ 1kb. The gel bands were cut from the gel under UV light using a sterile scalpel and extracted using the Qiagen QIAquick gel extraction kit.

## Ligation and transformation

The ligation of the samples into a plasmid was conducted using the Promega ‘pGEM®-T Easy Vector System 1’ as per the manufacturer's protocol and set up in the following reaction and left overnight at 4°C.

The transformation reaction used XL1-Blue competent cells (Agilent Technologies) and the manufactures ‘transformation protocol’ with some slight alterations. We added 2 µl of the ligated plasmid sample to the aliquot of cells (Step 5). We also used LB media instead of the suggested SOC media (step 9) and the LB-ampicillin plates were made by adding 15g of agar and 25g of LB medium to 1L of MiliQ water and mixing well. The LB agar was then autoclaved and left to cool to below 55°C. When the mixture reached the correct temperature, it was taken to a laminar flow cabinet and the filter-sterilized ampicillin was added (1 ml of a 100mg/ml solution). The agar is then mixed again and poured into Petri dishes and left to set in the laminar flow, under flame. For colour screening, 100µl of 100 mM IPTG and 20µl of 50 mg/ml X-gal was pipetted onto the agar and spread evenly (step 10). The plates were left for 48 hours for the colonies to develop as they were too small after the suggested 17 hours. After 48 hours of incubation at 37°C, the colonies were incubated at 4°C for 2 hours to enhance the colours. The growth of any bacteria that did not take up the plasmid would be inhibited by the antibiotic and if a colony formed and contained the plasmid but not the 16s gene we had tried to insert, then the colonies grow on the plates and appear blue in colour. Moreover, if the colonies contain the plasmid with the 16s gene insert, they appear white, making the colonies easy to sample. The colonies were sampled by touching them with a pipette tip and the tip was dropped into a falcon tube that contained 3ml of sterile LB-ampicillin media (same concentration of antibiotic as the plates). The sampled colonies were incubated at 37°C, with shaking at 225-250 rpm, overnight. The next day the plasmids were extracted from the sampled colonies using the Qiagen, QIAprep^®^ spin miniprep kit and quick-start protocol and eluted with nuclease-free water.

## Sample preparation for sequencing

Before the plasmids were sent for sequencing, 15 samples of each aphid line (each sample containing multiple aphids to obtain sufficient DNA yield) were digested with the restriction enzyme EcoR1. This enzyme removes the insert sequence from the plasmid we had used and enabled us to visually confirm its presence before sequencing. After digestion, when the samples were run on a 1% agarose gel, they showed two clear bands, one for the plasmid and a band of a similar size to the insert.

To prepare the samples for sequencing their concentration was checked on a Thermo Scientific™ nanodrop™ 2000 spectrophotometer. GATC Biotech AG, London UK, (now Eurofins GATC) recommended a DNA concentration of between 30 – 80 ng/µl for their plasmid ‘Supreme run’ Sanger sequencing. Those samples that had a concentration higher than recommended were then diluted, with nuclease-free water, to a final concentration of 60ng/µl. A total of 70 samples (35 Q1 and 35 N116) were sequenced using GATC Biotech’s T7 sequencing primers.

## Analysis of sequencing data

Sequences have been edited to remove pGEM-T Easy Vector sequences and any parts of the sequence that contained bases that did not pass the confidence threshold.

### N116 aphid symbiont BLAST analysis

Trimmed ‘N116’ clone *Acyrthosiphon pisum* symbiont 16s rRNA gene sequences used in the blast analysis shown in FASTA format. GenBank Accession numbers: MW979375 to MW979398

**>** **Seq1 [organism=Candidatus *Hamiltonella defensa*] 16S ribosomal RNA gene [host=*Acyrthosiphon pisum* isolate N116]**

AGAGTTTGATCCTGGCTCAGATTGAACACTGGTGGCAGGCCTAACACATGCAAGTCGAGCGGCATCGAGTGAGCGCAGTTTACTGAGTTCATGTCGGCGAGCGGCGGACGGGTGAGTAAAGTCTGGGAATCTGGCCGAAGGAGGGGGATAACTGCTGGAAACGGCAGCTAATACCGCATGAAGTCGCGAGACCAAAGTGGGGGACCTTCGGGCCTCACGCCTTCGGATGAGCCCAGATGAGATTAGCTGGTAGGTAAGGTAAGGGCTTACCTAGGCGACGATCTCTAGCGGGTCTGAGAGGATAGCCCGCCACACTGGAACTGAGACACGGTCCAGACTCCTACGGGAGGCAGCAGTGGGGAATATTGCACAATGGGCGAAAGCCTGATGCAGCCATGCCACGTGTGTGAAGAAGGCCTTCGGGTTGTAAAGCACTTTCAGCGAGGAGGAAGCGATAAATGCGAATACCATTTATTTTTGACGTTACTCGCAGAAGAAGCACCGGCTAACTCCGTGCCAGCAGCCGCGGTAATACGGAGGGTGCGAGCGTTGATCGGAATAACTGGGCGTAAAGGGCATGTAGGCGGTGAGTTAAGTCAGATGTGAAATCCCCGAGCTCAACTTGGGAATGGCATTTGAAACTGGGTCGCTAGAGTTTTCTAGAGGGGGGTAGAATTCCAGGTGTAGCGGTGAAATGCGTAGATATCTGGAGGAATACCGGTGGCGAAGGCGGCCCCCTGGAGAAAGACTGACGCTGAGGTGCGAAAGCGTGGGGAGCAAACAGGATTAGATACCCTGGTAGTCCACGCTGTAAACGATGTCGATTTGGAGGTTGCGGTCTTGAACTGTGGCGTCCGGAGCTAACGCG

**> Seq2 [organism=Candidatus *Fukatsuia symbiotica*] 16S ribosomal RNA gene [host=*Acyrthosiphon pisum* isolate N116]**

AGAGTTTGATCCTGGCTCAGATTGAACGCTGGCGGCAGGCCTAACACATGCAAGTCGAGCGGCATCGGGAAGGTAGCTTGCTATCTTTGCCGGCGAGCGGCGGACGGGTGAGTAAAGTCTGGGGATCTGCCTGATGGAGGGGGATAACTACTGGAAACGGTAGCTAATACCGCATGATGTTACGCGACCAAAGCGGGGGACCTCCGGGCCTCGCGCCATCAGATGAACCCAGATGGGATTAGCTAGTAGGAGAGGTAATGGCTCCCCTAGGCGACGATCCCTAGCTGGTCTGAGAGGATAACCAGCCACACTGGAACTGAGAGACGGTCCAGACTCCTACGGGAGGCAGCAGTGGGGAATATTGCACAATGGGCGCAAGCCTGATGCAGCCATGCCGCGTGTGTGAAGAAGGCCTTCGGGTTGTAAAGCACTTTCAGCGAGGAGGAATGAAGCAATGCAAAGAGTGTTGCTAATGGACGTTACTCGCAGAAGAAGCACCGGCTAACTCCGTGCCAGCAGCCGCGGTAATACGGAGGGTGCGAGCGTTAATCGGAATTACTGGGCATAAAGGGCACGTAGGCGGTTTCTTAAGTCAGATGTGAAATCCCCGAGCTTCACTTGGGAACGGCATTTGAAACTGAGAGTCTAGAGTTTTGTAGAGGGGGGTAGAATTCCAGGTGTAGCGGTGAAATGCGTAGATATCTGGAGGAATACCGGTGGCGAAGGCGGCCCCCTGGACAGAGACTGACGCTGAGGTGCGAAAGCGTGGGTAGCAAACAG

**> Seq3 [organism=Candidatus *Fukatsuia symbiotica*] 16S ribosomal RNA gene [host=*Acyrthosiphon pisum* isolate N116]**

AGAGTTTGATCCTGGCTCAGATTGAACGCTGGCGGCAGGCCTAACACATGCAAGTCGAGCGGCATCGGGAAGGTAGCTTGCTATCTTTGCCGGCGAGCGGCGGACGGGTGAGTAAAGTCTGGGGATCTGTCTGATGGAGGGGGATAACTACTGGAAACGGTAGCTAATACCGCATGATGTTACGCGACCAAAGCGGGGGACCTCCGGGCCTCGCGCCATCAGATGAACCCAGATGGGATTAGCTAGTAGGAGAGGTAATGGCTCCCCTAGGCGACGATCCCTAGCTGGTCTGAGAGGATAACCAGCCACACTGGAACTGAGAGACGGTCCAGACTCCTACGGGAGGCAGCAGTGGGGAATATTGCACAATGGGCGCAAGCCTGATGCAGCCATGCCGCGTGTGTGAAGAAGGCCTTCGGGTTGTAAAGCACTTTCAGCGAGGAGGAATGAAGCAATGCAAAGAGTGTTGCTAATGGACGTTACTCGCAGAAGAAGCACCGGCTAACTCCGTGCCAGCAGCCGCGGTAATACGGAGGGTGCGAGCGTTAATCGGAATTACTGGGCGTAAAGAGCACGTAGGCGGTTTCTTAAGTCAGATGTGAAATCCCCGAGCTTCACTTGGGAACGGCATTTGAAACTGAGAGTCTAGAGTTTTGTAGAGGGGGGTAGAATTCCAGGTGTAGCGGTGAAATGCGTAGATATCTGGAGGAATACCGGTGGCGAAGGCGGCCCCCTGGACAGAGACTGACGCTGAGGTGCGAAAGCGTGGGTAGCAAACAGGATTAGATACCCTGGTAGTCCACGCTGTAAACGATGTCGATTTGTAGGTTGTGGTTATAAACTGTGGCTTGCGGAGCAAACGCGTTAAATCGACCGCCTGGGGAGTACGGCCGCAAGGTTAAAACTCAAATGAATTGACGGGGGCCCGCACAAGCGGTGGAGCATGTGGTTTAATTCGATGCCACGCGAAGAACCTTACCTACTCTTGACATCCAGAGG

**> Seq4 [organism=Candidatus *Hamiltonella defensa*] 16S ribosomal RNA gene [host=*Acyrthosiphon pisum* isolate N116]**

AGAGTTTGATCCTGGCTCAGGTTGAACACTGGTGGCAGGCCTAACACATGCAAGTCGAGCGGCATCGAGTGAGCGCAGTTTACTGAGTTCATGTCGGCGAGCGGCGGACGGGTGAGTAAAGTCTGGGAATCTGGCCGAAGGAGGGGGATAACTGCTGGAAACGGCAGCTAATACCGCATGAAGTCGCGAGACCAAAGTGGGGGACCTTCGGGCCTCACGCCTTCGGATGAGCCCAGATGAGATTAGCTGGTAGGTAAGGTAAAGGCTTACCTAGGCGACGATCTCTAGCGGGTCTGAGAGGATAGCCCGCCACACTGGAACTGAGACACGGTCCAGACTCCTACGGGAGGCAGCAGTGGGGAATATTGCACAATGGGCGAAAGCCTGATGCAGCCATGCCACGTGTGTGAAGAAGGCCTTCGGGTTGTAAAGCACTTTCAGCGAGGAGGAAGCGATAAATGCGAATACCATTTATTTTTGACGTTACTCGCAGAAGAAGCACCGGCTAACTCCGTGCCAGCAGCCGCGGTAATACGGAGGGTGCGAGCGTTAATCGGAATAACTGGGCGTAAAGGGCATGTAGGCGGTGAGCTAAGTCAGATGTGAAATCCCCGAGCTCAACTTGGGAATGGCATTTGAAACTGGGTCGCTAGAGTTTTCTAGAGGGGGGTAGAATTCCAGGTGTAGCGGTGAAATGCGTAGATATCTGGAGGAATACCGGTGGCGAAGGCGGCCCCCTGGAGAAAGACTGACGCTGAGGTGCGAAAGCGTGGGGAGCAAACAGGATTAGATACCCTGGTAGTCCACGCTGTAANCGATGTCGATTTGGAGGTTGCGGTCTTGAACTGTGGCGTCCGGAGCTAACGCGTTAAATCGACCGCCTGGGGAGTACGGCCGCAAGGTTAAAACTCAAATGAATTGACGGGGGCCCGCACAAGCGGTGGAGCATGTGGTTTAATTCGATGCAACGCGAA

**> Seq5 [organism=Candidatus *Fukatsuia symbiotica*] 16S ribosomal RNA gene [host=*Acyrthosiphon pisum* isolate N116]**

AGAGTTTGATCCTGGCTCAGATTGAACGCTGGCGGCAGGCCTAACACATGCAAGTCGAGCGGCATCGGGAAGGTAGCTTGCTATCTTTGCCGGCGAGCGGCGGACGGGTGAGTAAAGTCTGGGGATCTGCCTGATGGAGGGGGATAACTACTGGAAACGGTAGCTAATACCGCATGATGTTACGCGACCAAAGCGGGGGACCTCCGGGCCTCGCGCCATCAGATGAACCCAGATGGGATTAGCTAGTAGGAGAGGTAATGGCTCCCCTAGGCGACGATCCCTAGCTGGTCTGAGAGGATAACCAGCCACACTGGAACTGAGAGACGGTCCAGACTCCTACGGGAGGCAGCAGTGGGGAATATTGCACAATGGGCGCAAGCCTGATGCAGCCATGCCGCGTGTGTGAAGAAGGCCTTCGGGTTGTAAAGCACTTTCAGCGAGGAGGAATGAAGCAATGCAAAGAGTGTTGCTAATGGACGTTACTCGCAGAAGAAGCACCGGCTAACTCCGTGCCAGCAGCCGCGGTAATACGGAGGGTGCGAGCGTTAATCGGAATTACTGGGCGTAAAGGGCACGTAGGCGGCTTCTTAAGTCAGATGTGAAATCCCCGAGCTTCACTTGGGAACGGCATTTGAAACTGAGAGTCTAGAGTTTTGTAGAGGGGGGTAGAATTCCAGGTGTAGCGGTGAAATGCGTAGATATCTGGAGGAATACCGGTGGCGAAGGCGGCCCCCTGGACAGAGACTGACGCTGAGGTGCGAAAGCGTGGGTAGCAANCAGGATTAGATACCCTGGTAGTCCACGCTGTAAACGATGTCGATTTGTAGGTTGTGGTTATAAACTGTGGCTTGCG

**> Seq6 [organism= Candidatus *Hamiltonella defensa*] 16S ribosomal RNA gene [host=*Acyrthosiphon pisum* isolate N116]**

AGAGTTTGATCCTGGCTCAGATTGAACACTGGTGGCAGGCCTAACACATGCAAGTCGAGCGGCATCGAGTGAGCGCAGTTTACTGAGTTCATGTCGGCGAGCGGCGGACGGGTGAGTAAAGTCTGGGAATCTGGCCGAAGGAGGGGGATAACTGCTGGAAACGGCAGCTAATACCGCATGAAGTCGCGAGACCAAAGTGGGGGACCTTCGGGCCTCACGCCTTCGGATGAGCCCAGATGAGATTAGCTGGTAGGTAAGGTAAAGGCTTACCTAGGCGACGATCTCTAGCGGGTCTGAGAGGATAGCCCGCCACACTGGAACTGAGACACGGTCCAGACTCCTACGGGAGGCAGCAGTGGGGAATATTGCACAATGGGCGAAAGCCTGATGCAGCCATGCCACGTGTGTGAAGAAGGCCTTCGGGTTGTAAAGCACTTTCAGCGAGGAGGAAGCGATAAATGCGAATACCATTTATTTTTGACGTTACTCGCAGAAGAAGCACTGGCTAACTCCGTGCCAGCAGCCGCGGTAATACGGAGGGTGCGAGCGTTAATCGGAATAACTGGGCGTAAAGGGCATGTAGGCGGTGAGTTAAGTCAGATGTGAAATCCCCGAGCTCAACTTGGGAATGGCATTTGAAACTGGGTCGCTAGAGTTTTCTAGAGGGGGGTAGAATTCCAGGTGTAGCGGTGAAATGCGTAGATATCTGGAGGAATACCGGTGGCGAAGGCGGCCCCCTGGAGAAAGACTGACGCTGAGGTGCGAAAGCGTGGGGAGCAAACAGGATTAGATACCCTGGTAGTCCACGCTGTAAACGATGTCGATTTGGAGGTTGCGGTCTTGAACTGTGGCGTCCGGAGCTAACGCGTTAAATCGACCGCCTGGGGAGTACGGCCGCAAGGTTAAAACTCAAATGAATTGACGGGGGCCCGCACAAGCGGTGGAGCATGTGGTTTAATTCGATGCAACGC

**>Seq7 [organism=Candidatus *Fukatsuia symbiotica*] 16S ribosomal RNA gene [host=*Acyrthosiphon pisum* isolate N116]**

GGTTACCTTGTTACGACTTCACCCCAGTCATGGTTCACAAAGTGGTAAGCGCCATCCCAAAGGTTAAGCTACCTACTTCTTTTGCAAAACATTCCCATGGTGTGACGGGCGGTGTGTACAAGGCCCGGGAACGTATTCACCGTAGCATTCTGATCCACGATTACTAGCGATTCCGACTTCATGGAGTCGAGTTGCAGACTCCAATCCGGACTACGACGTACTTTATGAGGTCCGCTCACCCTCGCAGGCTCGCTTCTCTTTGTATACGCCATTGTAGCACGTGTGTAGCCCTACTCGTAAGGGCCATGATGACTTGACGTCATCCCCACCTTCCTCCGGTTTATCACCGGCAGTCTCTCTTGAGTTCCCACCTCTACGTGCTGGCAACAAAAGATAAGGGTTGCGCTCGTTGCGGGACTTAACCCAACATTTCACAACACGAGCTGACGACAGCCATGCAGCACCTGTCTCAAAGCTCCCCGAAGGGCACGTCAACATCTCTGTCGACTCCTCTGGATGTCAAGAGTAGGTAAGGTTCTTCGCGTTGCATCGAATTAAACCACATGCTCCACCGCTTGTGCGGGCCCCCG

**> Seq9 [organism=Candidatus *Hamiltonella defensa*] 16S ribosomal RNA gene [host=*Acyrthosiphon pisum* isolate N116]**

AGAGTTTGATCCTGGCTCAGATTGAACACTGGTGGCAGGCCTAACACATGCAAGTCGAGCGGCATCGAGTGAGCGCAGTTTACTGAGTTCATGTCGGCGAGCGGCGGACGGGTGAGTAAAGTCTGGGAATCTGGCCGAAGGAGGGGGATAACTGCTGGAAACGGCAGCTAATACCGCATGAAGTCGCGAGACCAAAGTGGGGGACCTTCGGGCCTCACGCCTTCGGATGAGCCCAGATGAGATTAGCTGGTAGGTAAGGTAAAGGCTTACCTAGGCGACGATCTCTAGCGGGTCCGAGAGGATAGCCCGCCACACTGGAACTGAGACACGGTCCAGACTCCTACGGGAGGCAGCAGTGGGGAATATTGCACAATGGGCGAAAGCCTGATGCAGCCATGCCACGTGTGTGAAGAAGGCCTTCGGGTTGTAAAGCACTTTCAGCGAGGAGGAAGCGATAAATGCGAATACCATTTATTTTTGACGTTACTCGCAGAAGAAGCACCGGCTAACTCCGTGCCAGCAGCCGCGGTAATACGGAGGGTGCGAGCGTTAATCGGAATAACTGGGCGTAAAGGGCATGTAGGCGGTGAGTTAAGTCAGATGTGAAATCCCCGAGCTCAACTTGGGAATGGCATTTGAAACTGGGTCGCTAGAGTTTTCTAGAGGGGGGTAGAATTCCAGGTGTAGCGGTGAAATGCGTAGATATCTGGAGGAATACCGGTGGCGAAGGCGGCCCCCTGGAGAAAGACTGACGCTGAGGTGCGAAAGCGT

**> Seq10 [organism=Candidatus *Hamiltonella defensa*] 16S ribosomal RNA gene [host=*Acyrthosiphon pisum* isolate N116]**

AGAGTTTGATCCTGGCTCAGATTGAACACTGGTGGCAGGCCTAACACATGCAAGTCGAGCGGCATCGTGTGAGCGCAGTTTACTGAGTTCATGTCGGCGAGCGGCGGACGGGTGAGTAAAGTCTGGGAATCTGGCCGAAGGAGGGGGATAACTGCTGGAAACGGCAGCTAATACCGCATGAAGTCGCGAGACCAAAGTGGGGGACCTTCGGGCCTCACGCCTTCGGATGAGCCCAGATGAGATTAGCTGGTAGGTAAGGTAAAGGCTTACCTAGGCGACGATCTCTAGCGGGTCTGAGAGGATAGCCCGCCACACTGGAACTGAGACACGGTCCAGACTCCTACGGGAGGCAGCAGTGGGGAATATTGCACAATGGGCGAAAGCCTGATGCAGCCATGCCACGTGTGTGAAGAAGGCCTTCGGGTTGTAAAGCACTTTCAGCGAGGAGGAAGCGATAAATGCGAATACCATTTATTTTTGACGTTACTCGCAGAAGAAGCACCGGCTAACTCCGTGCCAGCAGCCGCGGTAATACGGAGGGTGCGAGCGTTAATCGGAATAACTGGGCGTAAAGGGCATGTAGGTGGTGAGTTAAGTCAGATGTGAAATCCCCGAGCTCAACTTGGGAATGGCATTTGAAACTGGGTCGCTAGGGTTTTCTAGGGGGGGTAGAATTCCAGGTGTAGCGGTGAAATGCGTAGATATCTGGAGGAATACCGGTGGCGAAGGCGGCCCCCTGGAGAAAGACTGACGCTGAGGTGCGAAAGCGTGGGGAGCAAACAGGATTAGATACCCTGGTAGTCCACGCTGTAAACGATGTCGATTTGGAGGTTGCGGTCTTGAACTGTGGCG

**> Seq11 [organism=Candidatus *Fukatsuia symbiotica*] 16S ribosomal RNA gene [host=*Acyrthosiphon pisum* isolate N116]**

AGAGTTTGATCCTGGCTCAGATTGAACGCTGGCGGCAGGCCTAACACATGCAAGTCGAGCGGCATCGGGAAGGTAGTTTGCTATCTTTGCCGGCGAGCGGCGGACGGGTGAGTAAAGTCTGGGGATCTGCCTGATGGAGGGGGATAACTACTGGAAACGGTAGCTAATACCGCATGATGTTACGCGACCAAAGCGGGGGACCTCCGGGCCTCGCGCCATCAGATGAACCCAGATGGGTTTAGCTAGTAGGAGAGGTAATGGCTCCCCTAGGCGACGATCCCTAGCTGGTCTGAGAGGATAACCAGCCACACTGGAACTGAGAGACGGTCCAGACTCCTACGGGAGGCAGCAGTGGGGAATATTGCACAATGGGCGCAAGCCTGATGCAGCCATGCCGCGTGTGTGAAGAAGGCCTTCGGGTTGTAAAGCACTTTCAGCGAGGAGGAATGAAGCAATGCAAAGAGTGTTGCTAATGGACGTTACTCGCAGAAGAAGCACCGGCTAACTCCGTGCCAGCAGCCGCGGTAATACGGAGGGTGCGAGCGTTAATCGGAATTACTGGGCGTAAAGAGCACGTAGGCGGTTTCTTAAGTCAGACGTGAAATCCCCGAGCTTCACTTGGGAACGGCATTTGAAACTGAGAGTCTAGAGTTTTGTAGAGGGGGGTAGAATTCCAGGTGTAGCGGTGAAATGCGTAGATATCTGGAGGAATACCGGTGGCGAAGGCGGCCCCCTGGACAGAGACTGACGCTGAGGTGCGAAAGCGTGGGTAGCAAACAGGATTAGATACCCTGGTAGTCCACGCTGTAAACGATGTCGATTTGTA

**> Seq12 [organism=Candidatus *Hamiltonella defensa*] 16S ribosomal RNA gene [host=*Acyrthosiphon pisum* isolate N116]**

AGAGTTTGATCCTGGCTCAGATTGAACACTGGTGGCAGGCCTAACACATACAAGTCGAGCGGCATCGAGTGAGCGCAGTTTACTGAGTTCATGTCGGCGAGCGGCGGACGGGTGAGTAAAGTCTGGGAATCTGGCCGAAGGAGGGGGATAACTGCTGGAAACGGCAGCTAATACCGCATGAAGTCGCGAGACCAAAGTGGGGGACCTTCGGGCCTCACGCCTTCGGATGAGCCCAGATGAGATTAGCTGGTAGGTAAGGTAAAGGCTTACCTAGGCGACGATCTCTAGCGGGTCTGAGAGGATAGCCCGCCACACTGGAACTGAGACACGGTCCAGACTCCTACGGGAGGCAGCAGTGGGGAATATTGCACAATGGGCGAAAGCCTGATGCAGCCATGCCACGTGTGTGAAGAAGGCCTTCGGGTTGTAAAGCACTTTCAGCGAGGAGGAAGCGATAAATGCGAATACCATTTATTTTTGACGTTACTCGCAGAAGAAGCACCGGCTAACTCCGTGCCAGCAGCCGCGGTAATACGGAGGGTGTGAGCGTTAATCGGAATAACTGGGCGTAAAGGGCATGTAGGCGGTGAGTTAAGTCAGATGTGAAATCCCCGAGCTCAACTTGGGAATGGCATTTGAAACTGGGTCGCTAGAGTTTTCTAGAGGGGGGTAGAATTCCAGGTGTAGCGGTGAAATGCGTAGATATCTGGAGGAATACCGGTGGCGAAGGCGGCCCCCTGGAGAAAGACTGACGCTGAGGTGCGAAAGC

**> Seq13 [organism=Candidatus *Hamiltonella defensa*] 16S ribosomal RNA gene [host=*Acyrthosiphon pisum* isolate N116]**

AGAGTTTGATCCTGGCTCAGATTGAACACTGGTGGCAGGCCTAACACATGCAAGTCGAGCGGCATCGAGTGAGCGCAGTTTACTGAGTTCATGTCGGCGAGCGGCGGACGGGTGAGTAAAGTCTGGGAATCTGGCCGAAGGAGGGGGATAACTGCTGGAAACGGCAGCTAATACCGCATGAAGTCGCGAGACCAAAGTGGGGGACCTTCGGGCCTCACGCCTTCGGATGAGCCCAGATGAGATTAGCTGGTAGGTAAGGTAAGGGCTTACCTAGGCGACGATCTCTAGCGGGTCTGAGAGGATAGCCCGCCACACTGGAACTGAGACACGGTCCAGACTCCTACGGGAGGCAGCAGTGGGGAATATTGCACAATGGGCGAAAGCCTGATGCAGCCATGCCACGTGTGTGAAGAAGGCCTTCGGGTTGTAAAGCACTTTCAGCGAGGAGGAAGCGATAAATGCGAATACCATTTATTTTTGACGTTACTCGCAGAAGAAGCACCGGCTAACTCCGTGCCAGCAGCCGCGGTAATACGGAGGGTGCGAGCGTTAATCGGAATAACTGGGCGTAAAGGGCATGTAGGCGGTGAGTTAAGTCAGATGTGAAATCCCCGAGCTCAACTTGGGAATGGCATTTGAAACTGGGTCGCTAGAGTTTTCTAGAGGGGGGTAGAATTCCAGGTGTAGCGGTGAAATGCGTAGATATCTGGAGGAATACCGGTGGCGAAGGCGGCCCCCTGGAGAAAGACTGACGCTGAGGTGCGAAAGCG

**> Seq14 [organism=Candidatus *Fukatsuia symbiotica*] 16S ribosomal RNA gene [host=*Acyrthosiphon pisum* isolate N116]**

GGTTACCTTGTTACGACTTCACCCCAGTCATGGTTCACAAAGTGGTAAGCGCCATCCCAAAGGTTAAGCTACCTACTTCTTTTGCAAAACACTCCCATGGTGTGACGGGCGGTGTGTACAAGGCCCGGGAACGTATTCACCGTAGCATTCTGATCCACGATTACTAGCGATTCCGACTTCATGGAGTCGAGTTGCAGACTCCAATCCGGACTACGACGTACTTTATGAGGTCCGCTCACCCTCGCAGGCTCGCTTCTCTTTGTATACGCCATTGTAGCACGTGTGTAGCCCTACTCGTAAGGGCCATGATGACTTGACGTCATCCCCACCTTCCTCCGGTTTATCACCGGCAGTCTCTCTTGAGTTCCCACCTCTACGTGCTGGCAACAAAAGATAAGGGTTGCGCTCGTTGCGGGACTTAACCCAACATTTCACAACACGAGCTGACGACAGCCATGCAGCACCTGTCTCAAAGCTCCCCGAAGGGCACGTCAACATCTCTGTCGACTCCTCTGGATGTCAAGAGTAGGTAAGGTTCTTCGCGTTGCATCGAATTAAACCACATGCTCCACCGCTTGTGCGGGCCCCCGTCAATTCATTTGAGTTTTAACCTTGCGGCCGTACTCCCCAGGCGGTCGATTTAACGCGTTTGCTCCGCAAGCCACAGTTTATAACCACAACCTACAAATCGACATCGTTTACAGCGTGGACTACCAGGGTATCTAATCCTGTTTGCTACCCTCGCTTTCGCACCTCAGCGTCAGTCTCTGTCCAGGGGGCCGCCTTCGCCACCGGTATTCCTCCAGATATCTACGCATTTCACCGCTACACCTGGAAATTCTACCCCCCCTCTACAAAACTCT

**> Seq15 [organism=** **Candidatus** ***Serratia symbiotica*] 16S ribosomal RNA gene [host=*Acyrthosiphon pisum* isolate N116]**

AGAGTTTGATCCTGGCTCAGATTGAACGCTGGCGGCAGGCCTAACACATGCAAGTCGAGCGGTAGCACAAGAGAGCTTGCTCTCTGGGTGACGAGCGGCGGACGGGTGAGTAATGTCTGGGAAACTGCCTGATGGCGGGGGATAACTAGTGGAAACGGTAGCTAATACCGCATAACGTCGCAAGACCAAAGTGGGGGACCTTCGGGCCTCACGCCATCAGATGTGCCCAGGTGGGATTAGCTGGTAGGTGGGGTAACGGCTCACCTAGGCGACGATCCCTAGCTGGTCTGAGAGGATGACCAGCCACACTGGAACTGAGACACGGTCCAGACTCCTACGGGAGGCAGCAGTGGGGAATATTGCACAATGGGCGCAAGCCTGATGCAGCCATGCCGCGTGTGTGAAGAAGGCCTTCGGGTTGTAAAGCACTTTCAGCGAGGAGAAAGGGTAATGTGTTAATAAGACATTGCATTGACGTTACTCGCAGAAGAAGCACCGGCTAACTCCGTGCCAGCAGCCGCGGTAATACGGAGGGTGCAAGCGTTAATCGGAATTACTGGGCGTAAAGCGCACGCAGGCGGTTTGTTAAGTCAGATGTGAAATCCCCGCGCTCAACGTAGGAACGGCATTTGAGACTGGCAAGCTAGAGTCTTGTAGAGGGGGGTAGAATTCCAGGTGTAGCGGTGAAATGCGTAGAGATCTGGAGGAATACCGGTGGCGAAGGCGGCCCCCTGGACAAAGACTGACGCTCAGGTGCGAAAGC

**> Seq16 [organism=Candidatus *Fukatsuia symbiotica*] 16S ribosomal RNA gene [host=*Acyrthosiphon pisum* isolate N116]**

GGTTACCTTGTTACGACTTCACCCCAGTCATGGTTCACAAAGTGGTAAGCGCCATCCCAAAGGTTAAGCTACCTACTTCTTTTGCAAAACACTCCCATGGTGTGACGGGCGGTGTGTACAAGGCCCGGGAACGTATTCACCGTAGCATTCTGATCCACGATTACTAGCGATTCCGACTTCATGGAGTCGAGTTGCAGACTCCAATCCGGACTACGACGTACTTTATGAGGTCCGCTCACCCTCGCAGGCTCGCTTCTCTTTGTATACGCCATTGTAGCACGTGTGTAGCCCTACTCGTAAGGGCCATGATGACTTGACGTCATCCCCACCTTCCTCCGGTTTATCACCGGCAGTCTCTCTTGAGTTCCCACCTCTACGTGCTGGCAACAAAAGATAAGGGTTGCGCTCGTTGCGGGACTTAACCCAACATTTCACAACACGAGCTGACGACAGCCATGCAGCACCTGTCTCAAAGCTCCCCGAAGGGCACGTCAACATCTCTGTCGACTCCTCTGGATGTCAAGAGTAGGTAAGGTTCTTCGCGTTGCATCGAATTAAACCACATGCTCCACCGCTTGTGCGGGCCCCCGTCAATTCATTTGAGTTTTAACCTTGCGGCCGTACTCCCCAGGCGGTCGATTTAACGCGTTTGCTCCGCAAGCCACAGTTTATAACCACAACCTACAAATCGACATCGTTTACAGCGTGGACTACCAGGGTATCTAATCCTGTTTGCTACCCACGCTTTCGCACCTCAGCGTCAGTCTCTGTCCAGGGGGG

**> Seq17 [organism=Candidatus *Hamiltonella defensa*] 16S ribosomal RNA gene [host=*Acyrthosiphon pisum* isolate N116]**

AGAGTTTGATCCTGGCTCAGATTGAACACTGGTGGCAGGCCTAACACATGCAAGTCGAGCGGCATCGAGTGAGCGCAGTTTACTGAGTTCATGTCGGCGAGCGGCGGACGGGTGAGTAAAGTCTGGGAATCTGGCCGAAGGAGGGGGATAACTGCTGGAAACGGCAGCTAATACCGCATGAAGTCGCGAGACCAAAGTGGGGGACCTTCGGGCCTCACGCCTTCGGATGAGCCCAGATGAGATTAGCTGGTAGGTAAGGTAAAGGCTTACCTAGGCGACGATCTCTAGCGGGTCTGAGAGGATAGCCCGCCACACTGGAACTGAGACACGGTCCAGACTCCTACGGGAGGCAGCAGTGGGGAATATTGCACAATGGGCGAAAGCCTGATGCAGCCATGCCACGTGTGTGAAGAAGGCCTTCGGGTTGTAAAGCACTTTCAGCGAGGAGGAAGCGATAAATGCGAATACCATTTATTTTTGACGTTACTCGCAGAAGAAGCACCGGCTAACTCCGTGCCAGCAGCCGCGGTAATACGGAGGGTGCGAGCGTTAATCGGAATAACTGGGCGTAAAGGGCATGTAGGCGGTGAGTTAAGTCAGATGTGAAATCCCCGAGCTCAACTTGGGAATGGCATTTGAAACTGGGTCGCTAGAGTTTTCTAGAGGGGGGTAGAATTCCAGGTGTAGCGGTGAAATGCGTAGATATCTGGAGGAATACCGGTGGCGAAGGCGGCCCCCTGGAGAAAGACTGACGCTGAGGTGCGAAAGC

**> Seq18 [organism=Candidatus *Fukatsuia symbiotica*] 16S ribosomal RNA gene [host=*Acyrthosiphon pisum* isolate N116]**

AGAGTTTGATCCTGGCTCAGATTGAACGCTGGCGGCAGGCCTAACACATGCAAGTCGAACGGCATCGGGAAGGTAGCTTGCTATCTTTGCCGGCGAGCGGCGGACGGGTGAGTAAAGTCTGGGGATCTGCCTGATGGAGGGGGATAACTACTGGAAACGGTAGCTAATACCGCATGATGTTACGCGACCAAAGCGGGGGACCTCCGGGCCTCGCGCCATCAGATGAACCCAGATGGGATTAGCTAGTAGGAGAGGTAATGGTTCCCCTAGGCGACGATCCCTAGCTGGTCTGAGAGGATAACCAGCCACACTGGAACTGAGAGACGGTCCAGACTCCTACGGGAGGCAGCAGTGGGGAATATTGCACAATGGGCGCAAGCCTGATGCAGCCATGCCGCGTGTGTGAAGAAGGCCTTCGGGTTGTAAAGCACTTTCAGCGAGGAGGAATGAAGCAATGCAAAGAGTGTTGCTAATGGACGTTACTCGCAGAAGAAGCACCGGCTAACTCCGTGCCAGCAGCCGCGGTAATACGGAGGGTGCGAGCGTTAATCGGAATTACTGGGCGTAAAGGGCACGTAGGCGGTTTCTTAAGTCAGATGTGAAATCCCCGAGCTTCACTTGGGAACGGCATTTGAAACTGAGAGTCTAGAGTTTTGTAGAGGGGGGTAGAATTCCAGGTGTAGCGGTGAAATGCGT

**> Seq19 [organism=Candidatus *Hamiltonella defensa*] 16S ribosomal RNA gene [host=*Acyrthosiphon pisum* isolate N116]**

AGAGTTTGATCCTGGCTCAGATTGAACACTGGTGGCAGGCCTAACACATGCAAGTCGAGCGGCATCGAGTGAGCGCAGTTTACTGAGTTCATGTCGGCGAGCGGCGGACGGGTGAGTAAAGTCTGGGAATCTGGCCGAAGGAGGGGGATAACTGCTGGAAACGGCAGCTAATACCGCATGAAGTCGCGAGACCAAAGTGGGGGACCTTCGGGCCTCACGCCTTCGGATGAGCCCAGATGAGATTAGCTGGTAGGTAAGGTAAAGGCTTACCTAGGCGACGATCTCTAGCGGGTCTGAGAGGATAGCCCGCCACACTGGAACTGAGACACGGTCCAGACTCCTACGGGAGGCAGCAGTGGGGAATATTGCACAATGGGCGAAAGCCTGATGCAGCCATGCCACGTGTGTGAAGAAGGCCTTCGGGTTGTAAAGCACTTTCAGCGAGGAGGAAGCGATAAATGCGAATACCATTTATTTTTGACGTTACTCGCAGAAGAAGCACCGGCTAACTCCGTGCCAGCAGCCGCGGTAATACGGAGGGTGCGAGCGTTAATCGGAATAACTGGGCGTAAAGGGCATGTAGGCGGTGAGTTAAGTCAGATGTGAAATCCCCGAGCTCAACTTGGGAATGGCATTTGAAACTGGGTCGCTAGAGTTTTCTAGAGGGGGGTAGAATTCCAGGTGTAGCGGTGAAATGCGTAGATATCTGGAGGAA

**> Seq21 [organism=Candidatus *Hamiltonella defensa*] 16S ribosomal RNA gene [host=*Acyrthosiphon pisum* isolate N116]**

AGAGTTTGATCCTGGCTCAGATTGAACACTGGTGGCAGGCCTAACACATGCAAGTCGAGCGGCATCGAGTGAGCGCAGTTTACTGAGTTCATGTCGGCGAGCGGCGGACGGGTGAGTAAAGTCTGGGAATCTGGCCGAAGGAGGGGGATAACTGCTGGAAACGGCAGCTAATACCGCATGAAGTCGCGAGACCAGAGTGGGGGACCTTTGGGCCTCACGCCTTCGGATGAGCCCAGATGTGATTAGCTGGTAGGTAAGGTAAAGGCTTACCTAGGCGACGATCTCTAGCGGGTCTGAGAGGATAGCCCGCCACACTGGAACTGAGACACGGTCCAGACTCCTACGGGAGGCAGCAGTGGGGAATATTGCCCAATGGGCGAAAGCCTGATGCAGCCATGCCACGTGTGTGAAGAAGGCCTTCGGGTTGTAAAGCACTTTCAGCGAGGAGGAAGCGATAAATGCGAATACCATTTATTTTTGACGTTACTCGCAGAAGAAGCACCGGCTAACTCCGTGCCAGCAGCCGCGGTAATACGGAGGGTGCGAGCGTTAATCGGAATAACTGGGCGTAAAGGGCATGTAGGCGGTGAGTTAAGCCAGATGTGAAATCCCCGAGCTCAACTTGGGAATGGCATTTGAAACTGGGTCGCTAGAGTTTTCTAGAGGGGGGTAGAATTCCAGGTGTAGCGGT

**> Seq22 [organism=Candidatus *Hamiltonella defensa*] 16S ribosomal RNA gene [host=*Acyrthosiphon pisum* isolate N116]**

AGAGTTTGATCCTGGCTCAGATTGAACACTGGTGGCAGGCCTAACACATGCAAGTCGAGCGGCATCGAGTGAGCGCAGTTTACTGAGTTCATGTCGGCGAGCGGCGGACGGGTGAGTAAAGTCTGGGAATCTGGCCGAAGGAGGGGGATAACTGCTGGAAACGGCAGCTAATACCGCATGAAGTCGCGAGACCAAAGTGGGGGACCTTCGGGCCTCACGCCTTCGGATGAGCCCAGATGAGATTAGCTGGTAGGTAAGGTAAAGGCTTACCTAGGCGACGATCTCTAGCGGGTCTGAGAGGATAGCCCGCCACACTGGAACTGAGACACGGTCCAGACTCCTACGGGAGGCAGCAGTGGGGAATATTGCACAATGGGCGAAAGCCTGATGCAGCCATGCCACGTGTGTGAAGAAGGCCTTCGGGTTGTAAAGCACTTTCAGCGAGGAGGAAGCGATAAATGCGAATACCATTTATTTTTGACGTTACTCGCAGAAGAAGCACCGGCTAACTCCGTGCCAGCAGCCGCGGTAATACGGAGGGTGCGAGCGTTAATCGGAATAACTGGGCGTAAAGGGCATGTAGGCGGTGAGTTAAGTCAGATGTGAAATCCCCGAGCTCAACTTGGGAATGGCATTTGAAACTGGGTCGCTAGAGTTTTCTAGAGGGGGGTAGAATTCCAGGTGTAGCGGTGAAATGCGTAGATATCTGGAGGAATACCGGTGGCGAAGGCGGCCCCCTGGAGAAAGACTGACGCTGAGG

**> Seq23 [organism=Candidatus *Hamiltonella defensa*] 16S ribosomal RNA gene [host=*Acyrthosiphon pisum* isolate N116]**

GGTTACCTTGTTACGACTTCACCCCAGTCATGAATCACAAAGTGGTAAGCGCCCTCCTTGCGGTTTAGCTACCTACTTCTTTTGCAACCCACTCCCATGGTGTGACGGGCGGTGTGTACAAGGCCCGGGAACGTATTCACCGTAGCATTCTGATCTACGATTACTAGCGATTCCGACTTCATGGAGTCGAGTTGCAGACTCCAATCCGGACTACGACATACTTTCTGAGTTCCGCTTTCCCTCGCAGGTTCGCATCCCTTTGTATACGCCATTGTAGCACGTGTGTAGCCCTACTCGTAAGGGCCATGATGACTTGACGTCGTCCCCACCTTCCTCCGGTTTATCACCGGCAGTCTCCTTTGAGTTCCCGCCTCTACGCGCTGGCAACAAAGGACAAGGGATGCGCTCGTTGCGGGACTTAACCCAACATTTCACAACACGAGCTGACGACAGCCATGCAGCACCTGTCTCACGGTTCCCGAAGGCACTTGCGCATCTCTGCACAATTCCGTGGATGTCAAGAGTAGGTAAGGTTCTTCGCGTTGCATCGAATTAAACCACATGCTCCACCGCTTGTGCGGGCCCCCGTCAATTCATTTGAGTTTTAACCTTGCGGCCGTACTCCCCAGGCGGTCGATTTAACGCGTTAGCTCCGGACGCCACAGTTCAAGACCGCAACCTCCAAATCGACATCGTTTACAGCGTGGACTACCAGGGTATCTAATCCTGTTTGCTCCCCACGCTTTCGCACCTCAGCGTCAGTCTTTCTCCAGGGGGCCGCCTTCGCCACCGGTATTCCTCCAGATATCTACGCATTTCACCGCTACACCT

**> Seq24 [organism=Candidatus *Fukatsuia symbiotica*] 16S ribosomal RNA gene [host=*Acyrthosiphon pisum* isolate N116]**

AGAGTTTGATCCTGGCTCAGATTGAACGCTGGCGGCAGGCCTAACACATGCAAGTCGAGCGGCATCGGGAAGGTAGCTTGCTATCTTTGCCGGCGAGCGGCGGACGGGTGAGTAAAGTCTGGGGATCTGCCTGATGGAGGGGGATAACTACTGGAAACGGTAGCTAATACCGCATGATGTTACGCGACCAAAGCGGGGGACCTCCGGGCCTCGCGCCATCAGATGAACCCAGATGGGATTAGCTAGTAGGAGAGGTAATGGCTCCCCTAGGCGACGATCCCTAGCTGGTCTGAGAGGATAACCAGCCACACTGGAACTGAGAGACGGTCCAGACTCCTACGGGAGGCAGCAGTGGGGAATATTGCACAATGGGCGCAAGCCTGATGCAGCCATGCCGCGTGTGTGAAGAAGGCCTTCGGGTTGTAAAGCACTTTCAGCGAGGAGGAATGAAGCAATGCAAAGAGTGTTGCTAATGGACGTTACTCGCAGAAGAAGCACCGGCTAACTCCGTGCCAGCAGCCGCGGTAATACGGAGGGTGCGAGCGTTAATCGGAATTACTGGGCGTAAAGGGCACGTAGGCGGTTTCTTAAGTCAGATGTGAAATCCCCGAGCTTCACTTGGGAACGGCATTTGAAACTGAGAGTCTAGAGTTTTGTAGAGGGGGGTAGAAT

**> Seq25 [organism=Candidatus *Hamiltonella defensa*] 16S ribosomal RNA gene [host=*Acyrthosiphon pisum* isolate N116]**

AGAGTTTGATCCTGGCTCAGATTGAACACTGGTGGCAGGCCTAACACATGCAAGTCGAGCGGCATCGAGTGAGCGCAGTTTACTGAGTTCATGTCGGCGAGCGGCGGACGGGTGAGTAAAGTCTGGGAATCTGGCCGAAGGAGGGGGATAACTGCTGGAAACGGCAGCTAATACCGCATGAAGTCGCGAGACCAAAGTGGGGGACCTTCGGGCCTCACGCCTTCGGATGAGCCCAGATGAGATTAGCTGGTAGGTAAGGTAAAGGCTTACCTAGGCGACGATCTCTAGCGGGTCTGAGAGGATAGCCCGCCACACTGGAACTGAGACACGGTCCAGACTCCTACGGGAGGCAGCAGTGGGGAATATTGCACAATGGGCGAAAGCCTGATGCAGCCATGCCACGTGTGTGAAGAAGGCCTTCGGGTTGTAAAGCACTTTCAGCGAGGAGGAAGCGATAAATGCGAATACCATTTATTTTTGACGTTACTCGCAGAAGAAGCACCGGCTAACTCCGTGCCAGCAGCCGCGGTAATACGGAGGGTGCGAGCGTTAATCGGAATAACTGGGCGTAAAGGGCATGTAGGCGGTGAGTTAAGTCAGATGTGAAATCCCCGAGCTCAACTTGGGAATGGCATTTGAAACTGGGTCGCTAGAGTTTTCTAGAGGGGGGTAGAATTCCAGGTGT

### Q1 aphid symbiont BLAST analysis

Trimmed ‘Q1 clone *Acyrthosiphon pisum* symbiont 16s rRNA gene sequences used in the blast analysis shown in FASTA format. GenBank Accession numbers: MW971996 to MW972018

**> Seq1 [organism=** **Candidatus** ***Serratia symbiotica*] 16S ribosomal RNA gene [host=*Acyrthosiphon pisum* isolate Q1]**

AGAGTTTGATCCTGGCTCAGATTGAACGCTGGCGGCAGGCCTAACACATGCAAGTCGAGCGGTAGCACAAGAGAGCTTGCTCTCTGGGTGACGAGCGGCGGACGGGTGAGTAATGTCTGGGAAACTGCCTGATGGCGGGGGATAACTAGTGGAAACGGTAGCTAATACCGCATAACGTCGCAAGACCAAAGTGGGGGACCTTCGGGCCTCACGCCATCAGATGTGCCCAGGTGGGATTAGCTGGTAGGTGGGGTAACGGCTCACCTAGGCGACGATCCCTAGCTGGTCTGAGAGGATGACCAGCCACACTGGAACTGAGACACGGTCCAGACTCCTACGGGAGGCAGCAGTGGGGAATATTGCACAATGGGCGCAAGCCTGATGCAGCCATGCCGCGTGTGTGAAGAAGGCCTTCGGGTTGTAAAGCACTTTCAGCGAGGAGAAAGGGTAATGTGTTAATAAGACATTGCATTGACGTTACTCGCAGAAGAAGCACCGGCTAACTCCGTGCCAGCAGCCGCGGTAATACGGAGGGTGCAAGCGTTAATCGGAATTACTGGGCGTAAAGCGCACGCAGGCGGTTTGTTAAGTCAGATGTGAAATCCCCGCGCTCAACGTAGGAACGGCATTTGAGACTGGCAAGCTAGAGTCTTGTAGAGGGGG

**> Seq2 [organism=** **Candidatus *Serratia symbiotica*] 16S ribosomal RNA gene [host=*Acyrthosiphon pisum* isolate Q1]**

AGAGTTTGATCCTGGCTCAGATTGAACGCTGGCGGCAGGCCTAACACATGCAAGTCGAGCGGTAGCACAAGAGAGCTTGCTCTCTGGGTGACGAGCGGCGGACGGGTGAGTAATGTCTGGGAAACTGCCTGATGGCGGGGGATAACTAGTGGAAACGGTAGCTAATACCGCATAACGTCGCAAGACCAAAGTGGGGGACCTTCGGGCCTCACGCCATCAGATGTGCCCAGGTAGGATTAGCTGGTAGGTGGGGTAACGGCTCACCTAGGCGACGATCCCTAGCTGGCCTGAGAGGATGACCAGCCACACTGGAACTGAGACACGGTCCAGACTCCTACGGGAGGCAGCAGTGGGGAATATTGCACAATGGGCGCAAGCCTGATGCAGCCATGCCGCGTGTGTGAAGAGGGCCTTCGGGTTGTAAAGCACTTTCAGCGAGGAGAAAGGGTAATGTGTTAATAAGACATTGCATTGACGTTACTCGCAGAAGAAGCACCGGCTAACTCCGTGCCTGCAGCCGCGGTAATACGGAGGGTGCAAGCGTTAATCGGAATTACTGGGCGTAAAGCGCACGCAGGCGGTTTGTTAAGTCAGATGTGAAATCCCCGCGCTCAACGTGGGAACGGCATTTGAGACTGGCAAGCTAGAGTCTTGTAGAGGGGGGTAGAATTC

**> Seq3 [organism=** **Candidatus *Serratia symbiotica*] 16S ribosomal RNA gene [host=*Acyrthosiphon pisum* isolate Q1]**

CGGCAGGCCTAACACATGCAAGTCGAGCGGTAGCACAAGAGAGCTTGCTCTCTGGGTGACGAGCGGCGGACGGGTGAGTAATGTCTGGGAAACTGCCTGATGGCGGGGGATAACTAGTGGAAACGGTAGCTAATACCGCATAACGTCGCAAGACCAAAGTGGGGGACCTTCGGGCCTCACGCCATCAGATGTGCCCAGGTGGGATTAGCTGGTAGGTGGGGTAACGGCTCACCTAGGCGACGATCCCTAGCTGGTCTGAGAGGATGACCAGCCACACTGGAACTGAGACACGGTCCAGACTCCTACGGGAGGCAGCAGTGGGGAATATTGCACAATGGGCGCAAGCCTGATGCAGCCATGCCGCGTGTGTGAAGAAGGCCTTCGGGTTGTAAAGCAC

**> Seq4 [organism=** **Candidatus *Serratia symbiotica*] 16S ribosomal RNA gene [host=*Acyrthosiphon pisum* isolate Q1]**

AGAGTTTGATCCTGGCTCAGATTGAACGCTGGCGGCAGGCCTAACACATGCAAGTCGAGCGGTAGCACAAGAGAGCTTGCTCTCTGGGTGACGAGCGGCGGACGGGTGAGTAATGTCTGGGAAACTGCCTGATGGCGGGGGATAACTAGTGGAAACGGTAGCTAATACCGCATAACGTCGCAAGACCAAAGTGGGGGACCTTCGGGCCTCACGCCATCAGATGTGCCCAGGTAGGATTAGCTGGTAGGTGGGGTAACGGCTCACCTAGGCGACGATCCCTAGCTGGTCTGAGAGGATGACCAGCCACACTGGAACTGAGACACGGTCCAGACTCCTACGGGAGGCAGCAGTGGGGAATATTGCACAATGGGCGCAAGCCTGATGCAGCCATGCCGCGTGTGTGAAGAAGGCCTTCGGGTTGTAAAGCACTTTCAGCGAGGAGAAAGGGTAATGTGTTAATAAGACATTGCATTGACGTTACTCGCAGAAGAAGCACCGGCTAACTCCGTGCCAGCAGCCGCGGTAATACGGAGGGTGCAAGCGTTAATCGGAATTACTGGGCGTAAAGCGCACGCAGGCGGTTTGTTAAGTCAGATGTGAAATCCCCGCGCTCAACGTGGGAACGGCATTTGAGACTGGCAAGCTAGAGTCTTGTAGAGGGGGGTAGAATT

**> Seq5 [organism=** **Candidatus *Serratia symbiotica*] 16S ribosomal RNA gene [host=*Acyrthosiphon pisum* isolate Q1]**

AGAGTTTGATCCTGGCTCAGATTGAACGCTGGCGGCAGGCCTAACACATGCAAGTCGAGCGGTAGCACAAGAGAGCTTGCTCTCTGGGTGACGAGCGGCGGACGGGTGAGTAATGTCTGGGAAACTGCCTGATGGCGGGGGATAACTAGTGGAAACGGTAGCTAATACCGCATAACGTCGCAAGACCAAAGTGGGGGACCTTCGGGCCTCACGCCATCAGATGTGCCCAGGTGGGATTAGCTGGTAGGTGGGGTAACGGCTCACCTAGGCGACGATCCCTAGCTGGTCTGAGAGGATGACCAGCCACACTGGAACTGAGACACGGTCCAGACTCCTACGGGAGGCAGCAGTGGGGAATATTGCACAATGGGCGCAAGCCTGATGCAGCCAGGCCGCGTGTGTGAAGAAGGCCTTCGGGTTGTAAAGCACTTTCAGCGAGGAGAAAGGGTAATGTGTTAATAAGACATTGCATTGACGTTACTCGCAGAAGAAGCACCGGCTAACTCCGTGCCAGCAGCCGCGGTAATACGGAGGGTGCAAGCGTTAATCGGAATTACTGGGCGTAAAGCGCACGCAGGCGGTTTGTTAAGTCAGATGTGAAATCCCCGCGCTCAACGTAGGAACGGCATTTGAGACTGGCAA

**> Seq6 [organism=** **Candidatus *Serratia symbiotica*] 16S ribosomal RNA gene [host=*Acyrthosiphon pisum* isolate Q1]**

AGAGTTTGATCCTGGCTCAGATTGAACGCTGGCGGCAGGCCTAACACATGCAAGTCGAGCGGTAGCACAAGAGAGCTTGCTCTCTGGGTGACGAGCGGCGGACGGGTGAGTAATGTCTGGGAAACTGCCTGATGGCGGGGGATAACTAGTGGAAACGGTAGCTAATACCGCATAACGTCGCAAGACCAAAGTGGGGGACCTTCGGGCCTCACGCCATCAGATGTGCCCAGGTGGGATTAGCTGGTAGGTGGGGTAACGGCTCACCTAGGCGACGATCCCTAGCTGGTCTGAGAGGATGACCAGCCACACTGGAACTGAGACACGGTCCAGACTCCTACGGGAGGCAGCAGTGGGGAATATTGCACAATGGGCGCAAGCCTGATGCAGCCATGCCGCGTGTGTGAAGAAGGCCTTCGGGTTGTAAAGCACTTTCAGCGAGGAGAAAGGGTAATGTGTTAATAAGACATTGCATTGACGTTACTCGCAGAAGAAGCACCGGCTAACTCCGTGCCAGCAGCCGCGGTAATACGGAGGGTGCAAGCGTTAATCGGAATTACTGGGCGTAAAGCGCACGCAGGCGGTTTGTTAAGTCAGATGTGAAATCCCCGCGCTCAACGTGGGAACGGCATTTGAGACTGGCAAGCTAGAGTCTTGTAGAGGGGGGTAGAATTCCAGGTGTAGCGGTGAAATGCG

**> Seq7 [organism=** **Candidatus *Serratia symbiotica*] 16S ribosomal RNA gene [host=*Acyrthosiphon pisum* isolate Q1]**

AGAGTTTGATCCTGGCTCAGATTGAACGCTGGCGGCAGGCCTAACACATGCAAGTCGAGCGGTAGCACAAGAGAGCTTGCTCTCTGGGTGACGAGCGGCGGACGGGTGAGTAATGTCTGGGAAACTGCCTGATGGCGGGGGATAACTAGTGGAAACGGTAGCTAATACCGCATAACGTCGCAAGACCAAAGTGGGGGACCTTCGGGCCTCACGCCATCAGATGTGCCCAGGTGGGATTAGCTGGTAGGTGGGGTAACGGCTCACCTAGGCGACGATCCCTAGCTGGTCTGAGAGGATGACCAGCCACACTGGAACTGAGACACGGTCCAGACTCCTACGGGAGGCAGCAGTGGGGAATATTGCACAATGGGCGCAAGCCTGATGCAGCCATGCCGCGTGTGTGAAGAAGGCCTTCGGGTTGTAAAGCACTTTCAGCGAGGAGAAAGGGTAATGTGTTAATAAGACATTGCATTGACGTTACTCGCAGAAGAAGCACCGGCTAACTCCGTGCCAGCAGCCGCGGTAATACGGAGG

**> Seq8 [organism=** ***Buchnera aphidicola*] 16S ribosomal RNA gene [host=*Acyrthosiphon pisum* isolate Q1]**

GGTTACCTTGTTACGACTTCACCCCAGTCATGAATCACAAAGTGGTAAGCGCCTTCCTTTTAAAGGGTTAGGATACCTGCTTCTTTTGCAACCCACTCCCATGGTGTGACGGGCGGTGTGTACAAGGCCCGGGAACGTATTCACCGTGGCATTCTGATCCACGATTACTAGCGATTCCGACTTCGTGGAGTCGAGTTGCAGACTCCAGTCCGGACTACGATTTACTTTATGAGGTTTGCTTGTCTTTGCAGATTTGCTTCTCTTTGTATAAACCATTGTAGCACGTGTGTAGCCCTGGTCGTAAGGGCCATGATGACTTGACGTCGTCCCCACCTTCCTCCGGTTTATAACCGGCAGTCTCCTCTGAGTTCCCGGCCGAACCGCTGGCAACAGGGGATAAGGGTTGCGCTCGTTGCGGGACTTAACCCAACATTTCACAACACGAGCTGACGACAGCCATGCAGCACCTGTCTCACAGCTCCCGAAGGCACTTCTTTATTTCTAAAGAATTCTGTGGATGTCAAGACCAGGTAAGGTTTTTCGCGTTGCATCGAATTAAACCACATGCTCCACCGCTTGTGCGGGCCCCCGTCAATTCATTTGAGTTTTAGCCTTGCGGCCGTACTCCCCAGGCGGTCGACTTAATGCGTTAGCTTCGGAAGTCACTTCTCTTGGAAACAACCTCCAAGTCGACATCGTTTACGGCATGGACCACCAGGGTATCTAATCCTGTTTGCTCCCCACGCTTTCGCGCCTCAGTGTCAGTTTTT

**> Seq9 [organism=** **Candidatus *Serratia symbiotica*] 16S ribosomal RNA gene [host=*Acyrthosiphon pisum* isolate Q1]**

AGAGTTTGATCCTGGCTCAGATTGAACGCTGGCGGCAGGCCTAACACATGCAAGTCGAGCGGTAGCACAAGAGAGCTTGCTCTCTGGGTGACGAGCGGCGGACGGGTGAGTAATGTCTGGGAAACTGCCTGATGGCGGGGGATAACTAGTGGAAACGGTAGCTAATACCGCATAACGTCGCAAGACCAAAGTGGGGGACCTTCGGGCCTCACGCCATCAGATGTGCCCAGGTAGGATTAGCTGGTAGGTGGGGTAACGGCTCACCTAGGCGACGATCCCTAGCTGGTCTGAGAGGATGACCAGCCACACTGGAACTGAGACACGGTCCAGACTCCTACGGGAGGCAGCAGTGGGGAATATTGCACAATGGGCGCAAGCCTGATGCAGCCATGCCGCGTGTGTGAAGAAGGCCTTCGGGTTGTAAAGCACTTTCAGCGAGGAGAAAGGGTAATGTGTTAATAAGACATTGCATTGACGTTACTCGCAGAAGAAGCACCGGCTAACTCCGTGCCAGCAGCCGCGGTAATACGGAGGGTGCAAGCGTTAATCGGAATTACTGGGCGTAAAGCGCACGCAGGCGGTTTGTTAAGTCAGATGTGAAATCCCCGCGCTCAACGTGGGAACGGCATTTTGAGACTG

**> Seq10 [organism=** **Candidatus *Serratia symbiotica*] 16S ribosomal RNA gene [host=*Acyrthosiphon pisum* isolate Q1]**

AGAGTTTGATCCTGGCTCAGATTGAACGCTGGCGGCAGGCCTAACACATGCAAGTCGAGCGGTAGCACAAGAGAGCTTGCTCTCTGGGTGACGAGCAGCGGACGGGTGAGTAATGTCTGGGAAACTGCCTGATGGCGGGGGATAACTAGTGGAAACGGTAGCTAATACCGCATAACGTCGCAAGACCAAAGTGGGGGACCTTCGGGCCTCACGCCATCAGATGTGCCCAGGTGGGATTAGCTGGTAGGTGGGGTAACGGCTCACCTAGGCGACGATCCCTAGCTGGTCTGAGAGGATGACCAGCCACACTGGAACTGAGACACGGTCCAGACTCCTACGGGAGGCAGCAGTGGGGAATATTGCACAATGGGCGCAAGCCTGATGCAGCCATGCCGCGTGTGTGAAGAAGGCCTTCGGGTTGTAAAGCACTTTCAGCGAGGAGAAAGGGTAATGTGTTAATAAGACATTGCATTGACGTTACTCGCAGAAGAAGCACCGGCTAACTCCGTGCCAGCAGCCGCGGTAATACGGAGGGTGCAAGCGTTAATCGGAATTACTGGGCGTAAAGCGCACGCAGGCGGTTTGTTAAGTCAGATGTGAAATCCCCGCGCTCAACGTGGGAACGGCATTTGAGACTGGCAAGCTAGAGTCTTGTAGAGGGGGGTAGAATTCCAGG

**> Seq11 [organism=** **Candidatus *Serratia symbiotica*] 16S ribosomal RNA gene [host=*Acyrthosiphon pisum* isolate Q1]**

AGAGTTTGATCCTGGCTCAGATTGAACGCTGGCGGCAGGCCTAACACATGCAAGTCGAGCGGTAGCACAAGAGAGCTTGCTCTCTGGGTGACGAGCGGCGGACGGGTGAGTAATGTCTGGGAAACTGCCTGATGGCGGGGGATAACTAGTGGAAACGGTAGCTAATACCGCATAACGTCGCAAGACCAAAGTGGGGGACCTTCGGGCCTCACGCCATCAGATGTGCCCAGGTGGGATTAGCTGGTAGGTGGGGTAACGGCTCACCTAGGCGACGATCCCTAGCTGGTCTGAGAGGATGACCAGCCACACTGGAACTGAGACACGGTCCAGACTCCTACGGGAGGCAGCAGTGGGGAATATTGCACAATGGGCGCAAGCCTGATGCAGCCATGCCGCGTGTGTGAAGAAGGCCTTCGGGTTGTAAAGCACTTTCAGCGAGGAGAAAGGGTAATGTGTTAATAAGACATTGCATTGACGTTACTCGCAGAAGAAGCACCGGCTAACTCCGTGCCAGCAGCCGCGGTAATACGGAGGGTGCAAGCGTTAATCGGAATTACTGGGCGTAAAGCGCACGCAGGCGGTTTGTTAAGTCAGATGTGAAATCCCCGCGCTCAACGT

**> Seq12 [organism=** **Candidatus *Serratia symbiotica*] 16S ribosomal RNA gene [host=*Acyrthosiphon pisum* isolate Q1]**

AGAGTTTGATCCTGGCTCAGATTGAACGCTGGCGGCAGGCCTAACACATGCAAGTCGAGCGGTAGCACAAGAGAGCTTGCTCTCTGGGTGACGAGCGGCGGACGGGTGAGTAATGTCTGGGAAACTGCCTGATGGCGGGGGATAACTAGTGGAAACGGTAGCTAATACCGCATAACGTCGCAAGACCAAAGTGGGGGACCTTCGGGCCTCACGCCATCAGATGTGCCCAGGTGGGATTAGCTGGTAGGTGGGGTAACGGCTCACCTAGGCGACGATCCCTAGCTGGTCTGAGAGGATGACCAGCCACACTGGAACTGAGACACGGTCCAGACTCCTACGGGAGGCAGCAGTGGGGAATATTGCACAATGGGCGCAAGCCTGATGCAGCCATGCCGCGTGTGTGAAGAAGGCCTTCGGGTTGTAAAGCACTTTCAGCGAGGAGAAAGGGTAATGTGTTAATAAGACATTGCATTGACGTTACTCGCAGAAGAAGCATCGGCTAACTCCGTGCCAGCAGCCGCGGTAATACGGAGGGTGCAAGCGTTAATCGGAATTACTGGGCGTAAAGCGCACGTAGGCGGTTTGTTAAGTCAGATGTGAAATCCCCGCGCTCAACGTGGGAACGGCATTTGAGACTGGCAAGCTAGAGTCTTGTAGAGGGGGGTAGAATTCCAGGTGTAGCGGTGAAATGCGTAGAGATCTGGAGGAATACCGGTGGCGAAGGCGGCCCCCTGGACAAAGACTGACGCTCAGGTGCGAAAGCGTGGGGAGCAAACAGGATTAGATACCCTGGTAGTCCACGCTGTAAACGATGTCGATTTGGAGGTTGCGCCCTTGAGGGGTGGCTTCCGTAGCTAACGCGTTAAATCGACCGCCTGGGGGAGTACG

**> Seq13 [organism=** **Candidatus *Serratia symbiotica*] 16S ribosomal RNA gene [host=*Acyrthosiphon pisum* isolate Q1]**

AGAGTTTGATCCTGGCTCAGATTGAACGCTGGCGGCAGGCCTAACACATGCAAGTCGAGCGGTAGCACAAGAGAGCTTGCTCTCTGGGTGACGAGCGGCGGACGGGTGAGTAATGTCTGGGAAACTGCCTGATGGCGGGGGATAACTAGTGGAAACGGTAGCTAATACCGCATAACGTCGCAAGACCAAAGTGGGGGACCTTCGGGCCTCACGCCATCAGATGTGCCCAGGTAGGATTAGCTGGTAGGTGGGGTAACGGCTCACCTAGGCGACGATCCCTAGCTGGTCTGAGAGGATGACCAGCCACACTGGAACTGAGACACGGTCCAGACTCCTACGGGAGGCAGCAGTGGGGAATATTGCACAATGGGCGCAAGCCTGATGCAGCCATGCCGCGTGTGTGAAGAAGGCCTTCGGGTTGTAAAGCACTTTCAGCGAGGAGAAAGGGTAATGTGTTAATAAGACATTGCATTGACGTTACTCGCAGAAGAAGCACCGGCTAACTCCGTGCCAGCAGCCGCGGTAATACGGAGGGTGCAAGCGTTAATCGGAATTACTGGGCGTAAAGCGCACGCAGGCGGTTTGTTAAGTCAGATGTGAAATCCCCGCGCTCAACGTGGGAACGGCATTTGAGACTGGCAAGCTAGAGTCTTGTAGAGGGGGGTAGAATTCCAGGTGTAGCGGTGAAATGCGTAGAGATCTGGAGGAATACCGGTGGCGAAGGCGGCCCCCTGGACAAAGACTGACGCTCAGGTGCGAAAGCGTGGGGAGCAAACAGGATTAGATACCCTGGTAGTCCACGCTGTAAACGATGTCGATTTGGAGGTTGCGCCCCTTG

**> Seq14 [organism=** **Candidatus *Serratia symbiotica*] 16S ribosomal RNA gene [host=*Acyrthosiphon pisum* isolate Q1]**

AGAGTTTGATCCTGGCTCAGATTGAACGCTGGCGGCAGGCCTAACACATGCAAGTCGAGCGGTAGCACAAGAGAGCCTGCTCTCTGGGTGACGAGCGGCGGACGGGTGAGTAATGTCTGGGAAACTGCCTGATGGCGGGGGATAACTAGTGGAAACGGTAGCTAATACCGCATAACGTCGCAAGACCAAAGTGGGGGACCTTCGGGCCTCACGCCATCAGATGTGCCCAGGTAGGATTAGCTGGTAGGTGGGGTAACGGCTCACCTAGGCGACGATCCCTAGCTGGTCTGAGAGGATGACCAGCCACACTGGAACTGAGACACGGTCCAGACTCCTACGGGAGGCAGCAGTGGGGAATATTGCACAATGGGCGCAAGCCTGATGCAGCCATGCCGCGTGTGTGAAGAAGGCCTTCGGGTTGTAAAGCACTTTCAGCGAGGAGAAAGGGTAATGTGTTAATAAGACATTGCATTGACGTTACTCGCAGAAGAAGCACCGGCTAACTCCGTGCCAGCAGCCGCGGTAATACGGAGGGTGCAAGCGTTAATCGGAATTACTGGGCGTAAAGCGCACGCAGGCGGTTTGTTAAGTCAGATGTGAAATCCCCGCGCTCAACGTGGGAACGGCATTTGAGACTGGCAAGCTAGAGTCTTGTAGAGGGGGGTAGAATTCCAGGTGTAGCGGTGAAATGCGTAGAGATCTGGAGGA

**> Seq15 [organism=** **Candidatus *Serratia symbiotica*] 16S ribosomal RNA gene [host=*Acyrthosiphon pisum* isolate Q1]**

AGAGTTTGATCCTGGCTCAGATTGAACGCTGGCGGCAGGCCTAACACATGCAAGTCGAGCGGTAGCACAAGAGAGCTTGCTCTCTGGGTGACGAGCGGCGGACGGGTGAGTAATGTCTGGGAAACTGCCTGATGGCGGGGGATAACTAGTGGAAACGGTAGCTAATACCGCATAACGTCGCAAGACCAAAGTGGGGGACCTTCGGGCCTCACGCCATCAGATGTGCCCAGGTGGGATTAGCTGGTAGGTGGGGTAACGGCTCACCTAGGCGACGATCCCTAGCCGGTCTGAGAGGATGACCAGCCACACTGGAACTGAGACACGGTCCAGACTCCTACGGGAGGCAGCAGTGGGGAATATTGCACAATGGGCGCAAGCCTGATGCAGCCATGCCGCGTGTGTGAAGAAGGCCTTCGGGTTGTAAAGCACTTTCAGCGAGGAGAAAGGGTAATGTGTTAATAAGACATTGCATTGACGTTACTCGCAGAAGAAGCACCGGCTAACTCCGTGCCAGCAGCCGCGGTAATACAGAGGGTGCAAGCGTTAATCGGAATTACTGGGCGTAAAGCGCACGCAGGCGGTTTGTTAAGTCAGATGTGAAATCCCCGCGCTCAACGTAGGAACGGCATTTGAGACTGGCAA

**> Seq16 [organism=** **Candidatus *Serratia symbiotica*] 16S ribosomal RNA gene [host=*Acyrthosiphon pisum* isolate Q1]**

AGAGTTTGATCCTGGCTCAGATTGAACGCTGGCGGCAGGCCTAACACATGCAAGTCGAGCGGTAGCACAAGAGAGCTTGCTCTCTGGGTGACGAGCGGCGGACGGGTGAGTAATGTCTGGGAAACTGCCTGATGGCGGGGGATAACTAGTGGAAACGGTAGCTAATACCGCATAACGTCGCAAGACCAAAGTGGGGGACCTTCGGGCCTCACGCCATCAGATGTGCCCAGGTGGGATTAGCTGGTAGGTGGGGTAACGGCTCACCTAGGCGACGATCCCTAGCTGGTCTGAGAGGATGACCAGCCACACTGGAACTGAGACACGGTCCAGACTCCTACGGGAGGCAGCAGTGGGGAATATTGCACAAAGGGCGCAAGCCTGATGCAGCCATGCCGCGTGTGTGAAGAAGGCCTTCGGGTTGTAAAGCACTTTCAGCGAGGAGAAAGGGTAATGTGTTAATAAGACATTGCATTGACGTTACTCGCA

**> Seq17 [organism= *Buchnera aphidicola*]** **16S ribosomal RNA gene [host=*Acyrthosiphon pisum* isolate Q1]**

AGAGTTTGATCCTGGCTCAGATTGAACGCTGGCGGCAAGCCTAACACATGCAAGTCGAGCGGCAGCGAGAAGAGAGCTTGCTCTCTTTGTCGGCAAGCGGCAAACGGGTGAGTAATATCTGGGGATCTACCCAAAAGAGGGGGATAACTACTAGAAATGGTAGCTAATACCGCATAATGTTGAAAAACCAAAGTGGGGGACCTTTTGGCCTCATGCTTTTGGATGAACCCAGACGAGATTAGCTTGTTGGTAGAGTAATAGCCTACCAAGGCAACGATCTCTAGCTGGTCTGAGAGGATAACCAGCCACACTGGAACTGAGACACGGTCCAGACTCCTACGGGAGGCAGCAGTGGGGAATATTGCACAATGGGCGAAAGCCTGATGCAGCTATGCCGCGTGTATGAAGAAGGCCTTAGGGTTGTAAAGTACTTTCAGCGGGGAGGAAAAAAATAAAACTAATAATTTTATTTCGTGACGTTACCCGCAGAAGGAGCACCGGCTAACTCCGTGCCAGCAGCCGCGGTAATACGGAGGGTGCAAGCGTTAATCAGAATTACTGGGCGTAAAGAGCGCGTAGGTGGTTTTTTAAGTCAGGTGTGAAATCCCTAGGCTCAACCTAGGAACTGCATTTGAAACTGGAAAACTAGAGTTTCGTAGAGGGAGGTAGAATTCTAGGTGTAGCGGTGAAATGCGTAGATATCTGGAGGAATACCCGTGGCGAAAGCGGCCTCCTAAACGAAAACTGACACTGAGGCGCGAAAGCGTGGGGAGCAAACAGGATTAGATACCCTGGTAGTCCATGCCGTAAACGATGTCGACTTGGAGGTTGTTTCCAAGAGAAGTGACTTCCGAAGCTAACGCATTAAGTCGACCGCCTGGGGGAGTACGGCCGCAAGGCTAAAACTCAAATGAATTGACGGGGGCCCGCACAAGC

**> Seq18 [organism= *Buchnera aphidicola*]** **16S ribosomal RNA gene [host=*Acyrthosiphon pisum* isolate Q1]**

AGAGTTTGATCCTGGCTCAGATTGAACGCTGGCGGCAAGCCTAACACATGCAAGTCGAGCGGCAGCGAGAAGAGAGCTTGCTCTCTTTGTCGGCAAGCGGCAAACGGGTGAGTAATATCTGGGGATCTACCCAAAAGAGGGGGATAACTACTAGAAATGGTAGCTAATACCGCATAATGTTGAAAAACCAAAGTGGGGGACCTTTTGGCCTCATGCTTTTGGATGAACCCAGACGAGATTAGCTTGTTGGTAGAGTAATAGCCTACCAAGGCAACGATCTCTAGCTGGTCTCAGAGGATAACCAGCCACACTGGAACTGAGACACGGTCCAGACTCCTACGGGAGGCAGCAGTGGGGAATATTGCACAATGGGCGAAAGCCTGATGCAGCTATGCCGCGTGTATGAAGAAGGCCTTAGGGTTGTAAAGTACTTTCAGCGGGGAGGAAAAAAATAAAACTAATAATTTTATTTCGTGACGTTACCCGCAGAAGAAGCACCGGCTAACTCCGTGCCAGCAGCCGCGGTAATACGGAGGGTGCAAGCGTTAATCAGAATTACTGGGCGTAAAGAGCGCGTAGGTGGTTTTTTAAGTCAGGTGTGAAATCCCTAGGCTCAACCTAGGAACTGCACTTGAAACTGGAAAACTAGAGTTTCGTAGAGGGAGGTAGAATTCTAGGTGTAGCGGTGAAATGCGTAGATATCTGGAGGAATACCCGTGGCGAAAGCGGCCTCCTAAACGAAAACTGACACTGAGGCGCGAAAGCGTGGGGAGCAAACAG

**> Seq19 [organism= Candidatus *Serratia symbiotica*]** **16S ribosomal RNA gene [host=*Acyrthosiphon pisum* isolate Q1]**

AGAGTTTGATCCTGGCTCAGATTGAACGCTGGCGGCAGGCCTAACACATGCAAGTCGAGCGGTAGCACAAGAGAGCTTGCTCTCTGGGTGACGAGCGGCGGACGGGTGAGTAATGTCTGGGAAACTGCCTGATGGCGGGGGATAACTAGTGGAAACGGTAGCTAATACCGCGTAACGTCGCAAGACCAAAGTGGGGGACCTTCGGGCCTCACGCCATCAGATGTGCCCAGGTGGGATTAGCTGGTAGGTGGGGTAACGGCTCACCTAGGCGACGATCCCTAGCTGGTCTGAGAGGATGACCAGCCACACTGGAACTGAGACACGGTCCAGACTCCTACGGGAGGCAGCAGTGGGGAATATTGCACAATGGGCGCAAGCCTGATGCAGCCATGCCGCGTGTGTGAAGAAGGCCTTCGGGTTGTAAAGCACTTTCAGCGAGGAGAAAGGGTAATGTGTTAATAAGACATTGCATTGACGTTACTCGCAGAAGAAGCACCGGCTAACTCCGTGCCAGCAGCCGCGGTAATACGGAGGGTGCAAGCGTTAATCGGAATTACTGGGCGTAAAGCGCACGCAGGCGGTTTGTTAAGTCAGATGTGAAATCCCCGCGCTCAACGTGGGAACGGCATTTGAGACTGGCAAGCTAGAGTCTTGCAGAGGGGGGTAGAATTCCAGGTGTAGCGGTGAAATGCGTAGAGATCTGGAGGAATACCGGTGGCGAAGGCGGCCCCCAGGACAAAGACTGACGCTCAGGTGCGAAAGCGTGGGGAGCAAACAGGATTAGATACCCTGGTAGTCCACGCTGTAAACGATGTCGATTTGGAGGTTGCGCCCTTGAGGGGTGGCTTCCGTAGCTAACGCGTTAAATCGACCGCCTGGGGAGTACGGCCGcAAGGTTAAAACTCAAATGAATTGACGGGGGCCCGCACAAGCGGTGGAGCATGTGGTTTAATTCGATGCAACGCG

**> Seq20 [organism= Candidatus *Serratia symbiotica*]** **16S ribosomal RNA gene [host=*Acyrthosiphon pisum* isolate Q1]**

AGAGTTTGATCCTGGCTCAGATTGAACGCTGGCGGCAGGCCTAACACATGCAAGTCGAGCGGTAGCACAAGAGAGCTTGCTCTCTGGGTGACGAGCGGCGGACGGGTGAGTAATGTCTGGGAAACTGCCTGATGGCGGGGGATAACTAGTGGAAACGGTAGCTAATACCGCATAACGTCGCAAGACCAAAGTGGGGGACCTTCGGGCCTCACGCCATCAGATGTGCCCAGGTAGGATTAGCTGGTAGGTGGGGTAACGGCTCACCTAGGCGACGATCCCTAGCTGGTCTGAGAGGATGACCAGCCACACTGGAACTGAGACACGGTCCAGACTCCTACGGGAGGCAGCAGTGGGGAATATTGCACAATGGGCGCGAGCCTGATGCAGCCATGCCGCGTGTGTGAAGAAGGCCTTCGGGTTGTAAAGCACTTTCAGCGAGGAGAAAGGGTAATGTGTTAATAAGACATTGCATTGACGTTACTCGCAGAAGAAGCACCGGCTAGCTCCGTGCCAGCAGCCGCGGTAATACGGAGGGTGCAAGCGTTAATCGGAATTACTGGGCGTAAAGCGCACGCAGGCGGTTTGTTAAGTCAGATGTGAAATCCCCGCGCTCAACGTGGGAACGGCATTTGAGACTGGCAAGCTAGAGTCTTGTAGAGGGGGGTAGAATTCCAGGTGTAGCGGTGAAATGCGTAGAGATCTGGAGGAATACCGGTGGCGAAGGCGGCCCCCTGGACAAAGACTGACGCTCAGGTGCGAAAGCGTGGGGAGCAAACAGGATTAGATACCCTGGTAGTCCACGCTGTAAACGATGTCGATTTGGAGGTTGCGCCCTTGAGGGGTGGCTTCCGTAGCTAACGCGTTAAATCGACCGCC

**> Seq21 [organism= Candidatus *Serratia symbiotica*]** **16S ribosomal RNA gene [host=*Acyrthosiphon pisum* isolate Q1]**

AGAGTTTGATCCTGGCTCAGATTGAACGCTGGCGGCAGGCCTAACACATGCAAGTCGAGCGGTAGCACAAGAGAGCTTGCTCTCTGGGTGACGAGCGGCGGACGGGTGAGTAATGTCTGGGAAACTGCCTGATGGCGGGGGATAACTAGTGGAAACGGTAGCTAATACCGCATAACATCGCAAGACCAAAGTGGGGGACCTTCGGGCCTCACGCCATCAGATGTGCCCAGGTAGGATTAGCTGGTAGGTGGGGTAACGGCTCACCTAGGCGACGATCCCTAGCTGGTCTGAGAGGATGACCAGCCACACTGGAACTGAGACACGGTCCAGACTCCTACGGGAGGCAGCAGTGGGGAATATTGCACAATGGGCGCAAGCCTGATGCAGCCATGCCGCGTGTGTGAAGAAGGCCTTCGGGTTGTAAAGCACTTTCAGCGAGGAGAAAGGGTAATGTGTTAATAAGACATTGCATTGACGTTACTCGCAGAAGAAGCACCGGCTAACTCCGTGCCAGCAGCCGCGGTAATACGGAGGGTGCAAGCGTTAATCGGAATTACTGGGCGTAAAGCGCACGCAGGCGGTTTGTTAAGTCAGATGTGAAATCCCCGCGCTCAACGTGGGAACGGCATTTGAGACTGGCAAGCTAGAGTCTTGTAGAGGGGGGTAGAATTCCAGGTGTAGCGGTGAAATGCGTAGAGATCTGGAGGAATACCGGTGGCGAAGGCGGCCCCCTGGACAAAGACTGACGCTCAGGTGCGAAAGCGTGGGGAGCAAACAGGATTAGATACCCTGGTAGTCCACGCTGTAAACGATGTCGATTTGGAGGTTGCGCCCTTGAGGGGTGGCTTCCGTAGCTAACGCGTTAAATCGACCGCCTGGGGAGTACGGCCGCAAGGTTAAAACTCAAATGAATTGACGGGGGCCCGCACAAGC

**> Seq22 [organism= Candidatus *Serratia symbiotica*]** **16S ribosomal RNA gene [host=*Acyrthosiphon pisum* isolate Q1]**

AGAGTTTGATCCTGGCTCAGATTgaACGCTGGCGGCAGGCCTAACACATGCAAGTCGAGCGGTAGCACAAGAGAGCTTGCTCTCTGGGTGACGAGCGGCGGACGGGTGAGTAATGTCTGGGAAACTGCCTGATGGCGGGGGATAACTAGTGGAAACGGTAGCTAATACCGCATAACGTCGCAAGACCAAAGTGGGGGACCTTCGGGCCTCACGCCATCAGATGTGCCCAGGTGGGATTAGCTGGTAGGTGGGGTAACGGCTCACCTAGGCGACGATCCCTAGCTGGTCTGAGAGGATGACCAGCCACACTGGAACTGAGACACGGTCCAGACTCCTACGGGAGGCAGCAGTGGGGAATATTGCACAATGGGCGCAAGCCTGATGCAGCCATGCCGCGTGTGTGAAGAAGGCCTTCGGGTTGTAAAGCACTTACAGCGAGAAGAAAGGGTAATGTGTTAATAAGACATTGCATTGACGTTACTCGCAGAAGAAGCACCGGCTAACTCCGTGCCAGCAGCCGCGGTAATACGGAGGGTGCAAGCGTTAATCGGAATTACTGGGCGTAAAGCGCACGCAGGCGGTTTGTTAAGTCAGATGTGAAATCCCCGCGCTCAACGTAGGAACGGCATTTGAGACTGGCAAGCTAGAGTCTTGTAGAGGGGGGTAGAATTCCAGGCGTAGCGGTGAAATGCGTAGAGATCTGGAGGAATACCGGTGGCGAAGGCGGCCCCCTGGACAAAGACTGACGCTCAGGTGCGAAAGCGTGGGGAGCAAACAGGATTAGATACCCTGGTAGTCCACGCTGTAAACGATGTCGATTTGGAGGTTGCGCCCTTGAGGGGTGGCTTCCGTAGCTAACGCGTTAAATCGACCGCCTGGGGAGTACGGCCGCAAGGTTAAAACTCAAATGAATTGACGGGGGCCCGCACAAGCGGTGGaGCATGTGGTTTAATTCGATGCAACGCGA

**> Seq23 [organism= Candidatus *Serratia symbiotica*]** **16S ribosomal RNA gene [host=*Acyrthosiphon pisum* isolate Q1]**

GGCCTAACACATGCAAGTCGAGCGGTAGCACAAGAGAGCTTGCTCTCTGGGTGACGAGCGGCGGACGGGTGAGTAATGTCTGGGAAACTGCCTGATGGCGGGGGATAACTAGTGGAAACGGTAGCTAATACCGCATAACGTCGCAAGACCAAAGTGGGGGACCTTCGGGCCTCACGCCATCAGATGTGCCCAGGTAGGATTAGCTGGTAGGTGGGGTAACGGCTCACCTAGGCGACGATCCCTAGCTGGTCTGAGAGGATGACCAGCCACACTGGAACTGAGACACGGTCCAGACTCCTACGGGAGGCAGCAGCGGGGAATATTGCACAATGGGCGCAAGCCTGATGCAGCCATGCCGCGTGTGTGAAGAAGGCCTTCGGGTTGTAAAGCACCTTCAGCGAGGAGAAAGGGTAATGTGTTAATAAGACATTGCATTGACGTTACTCGCAGAAGAAGCACCGGCTAACTCCGTGCCAGCAGCCGCGGTAATACGGAGGGTGCAAGCGTTAATCGGAATTACTGGGCGTAAAGCGCACGCAGGCGGTTTGTTAAGTCAGATGTGAAATCCCCGCGCTCAACGGGGGAACGGCATTTGAGACTGGCAAGCTAGAGTCTTGTAGAGGGGGGTAGAATTCCAGGTGTAGCGGTGAAATGCGTAGAGATCTGGAGGAATACCGGTGGCGAAGGCGGCCCCCTGGACAAAGACTGACGCTCAGGTGCGAAAGCGTGGGGAGCAAACAGGATTAGATACCCTGGTAGTCCACGCTGTAAACGATGTCGATTTGGAGGTTGCGCCCTTGAGGGGTGGCTTCCGTAGCTAACGCGTTAAATCGACCGCCTGGGGAGTACGGCCGCAAGGTTAAAACTCAAATGAATTGACGGGGGCCCGCACAAGCGGTGGAGCATGT
